# Beyond additive effects: examining the combined impact of air pollutant interactions on pulmonary tuberculosis in China

**DOI:** 10.1186/s12889-025-24421-5

**Published:** 2025-09-24

**Authors:** Zhe Pang, Xineng Jiang, Kui Liu, Yaling Feng, Jing Wei, Bin Chen, Bo Xie

**Affiliations:** 1 School of Urban Design, Wuhan University, Wuhan, 430072 China; 2 Lanxi County Center for Disease Control and Prevention, Zhejiang 321100 Jinhua, China; 3 Zhejiang Provincial Center for Disease Control and Prevention, Hangzhou, 310051 China; 4 MEEKL-AERM, College of Environmental Sciences and Engineering, Institute of Tibetan Plateau, and Center for Environment and Health, Peking University, 100871 Beijing, China; 5 Department of Atmospheric and Oceanic Science, Earth System Science Interdisciplinary Center, University of Maryland, 20740 College Park, USA

**Keywords:** Air pollutant mixture, Greenness, Pulmonary tuberculosis, Interactive effect, Additive effect, Case–control study

## Abstract

**Background:**

Various ambient air pollutants within a mixture may interact with each other and amplify or reduce the cumulative effects of individual air pollutants on health outcomes. However, health-related studies on the interactive effects of air pollutant mixtures remain limited. Additionally, the influence of greenness on health outcomes in the context of air pollutant mixtures has seldom been explored.

**Objective:**

To develop a joint analysis framework that focuses on the interactive effects among pollutants to evaluate the combined effects of ambient air pollutant mixtures on pulmonary tuberculosis (PTB) risks, taking into account greenness as a moderating factor.

**Methods:**

In this case–control study conducted in Lanxi, China, 1128 newly diagnosed PTB cases from 2019 to 2021 were matched with 2256 controls by sex and age. To evaluate exposure, the 24-month average values of particulate matter with aerodynamic diameters of ≤ 2.5 µm (PM_2.5_), sulfur dioxide (SO_2_), ozone (O_3_), nitrogen dioxide (NO_2_), and the Normalized Difference Vegetation Index before diagnosis or study entry were assessed using a high-resolution dataset. A quantile-based g-computation model was then used to estimate the additive and interactive effects of air pollutants on PTB risks and identify the moderating influence of greenness on these relationships.

**Results:**

Additive effect models showed that a one quartile increase in exposure to the air pollutant mixture was associated with elevated PTB risks (mixture odds ratio: 1.17, 95% confidence intervals: 1.07, 1.36), with NO_2_ contributing the most significant positive effect. Interactive effect models showed that incorporating interaction terms for O_3_ and other pollutants resulted in PTB risks that exceeded those estimated using the additive effects of various pollutants. Furthermore, areas with higher levels of greenness exhibited lower PTB risks associated with air pollutant mixture than areas with lower levels of greenness.

**Conclusions:**

To reduce biases in air pollution control policies and maximize their health benefits, it is essential to assess both the additive and interactive effects when evaluating the health impacts of air pollutant mixtures. Furthermore, the influence of greenness should be considered in the context of these air pollutant mixtures.

**Supplementary Information:**

The online version contains supplementary material available at 10.1186/s12889-025-24421-5.

## Introduction

Ambient air pollution is a major contributor to the global disease burden, responsible for 4.5 million deaths worldwide [1]. Among the various health impacts of air pollution, recent laboratory-based studies have specifically highlighted that exposure to fine particles and gaseous pollutants could accelerate the infection and activation of *Mycobacterium tuberculosis* (*M.tb*) [2], leading to active pulmonary tuberculosis (PTB) [3]. Empirical evidence further supports these findings, showing that long-term exposure to elevated levels of particulate matter with aerodynamic diameter of ≤ 2.5 µm (PM_2.5_), nitrogen dioxide (NO_2_), sulfur dioxide (SO_2_), and ozone (O_3_) is associated with an increased risk of PTB [4, 5]. While these epidemiological studies have linked individual pollutants to health outcomes such as PTB risk [4, 5], they do not fully capture real-world scenarios where individuals are often exposed to a mixture of pollutants. Evaluating the impact of specific pollutants in isolation may not offer a strong basis for developing effective public health interventions. Recognizing this limitation, some researchers have shifted their focus from studying individual pollutants to examining air pollutant mixtures within a joint framework. For instance, several studies have explored the relationship between long-term exposure to air pollutant mixtures (comprising three or more components) and the incidence or mortality of nonaccidental deaths [6, 7], cardiovascular diseases [8, 9], and respiratory diseases [6, 8], consistently identifying harmful effects.

However, these studies often rely solely on additive models, such as cumulative risk indices [6, 10] and air pollution scores [11, 12]. While these models account for the cumulative or offsetting effects between air pollutants, they often overlook the potential additional impacts that arise from complex interactions within pollutant mixtures. Specifically, gases such as NO_2_, which are usually filtered by the upper respiratory system, may attach to particulate matter such as PM_2.5_, allowing them to penetrate deeper into the lungs [13]. Additionally, chemical interactions between gaseous and particulate pollutants within complex air pollutant mixtures can lead to the formation of secondary pollutant components, such as sulfuric acids and secondary organic aerosols that contribute to secondary PM_2.5_ components [14, 15]. These new components may have greater oxidative potential and pro-inflammatory properties, which can aggravate pulmonary inflammation [16, 17], potentially increase susceptibility to *M.tb*. Nevertheless, it remains unclear whether these interactive effects of various pollutants would influence the risks of PTB and whether they amplify these risks beyond the cumulative effects of individual pollutants.

Moreover, while greenness has been shown to affect levels of individual air pollutants and their associated health risks [18–21], their role in modifying the health effects of complex pollutant mixtures remains underexplored. The potential mechanisms by which greenness moderates the relationship between air pollutant mixture and disease risks may differ from those observed with single pollutants. Greenness may modify the environmental context of air pollutant mixtures by influencing how pollutants co-occur and interact. These changes in exposure patterns may affect immune responses to *M.tb* [22, 23], potentially contributing to spatial differences in the health effects of air pollutant mixture across areas with varying levels of greenness. However, studies focusing on individual pollutants are limited in their ability to capture how greenness shapes the combined effects of multiple pollutants.

To address these gaps, we conducted a population-based case–control study in Lanxi City, China. The objectives were 1) to develop a joint analysis framework that assesses both the additive effects (i.e., without interaction terms) and interactive effects (i.e., with interaction terms) of air pollutants within the mixture on PTB risks and 2) to examine the moderating effect of greenness on the relationship between air pollutant mixtures and PTB risks within 250 m, 500 m, and 1000 m buffers.

## Materials and methods

### Study design and population

**Fig. 1:**
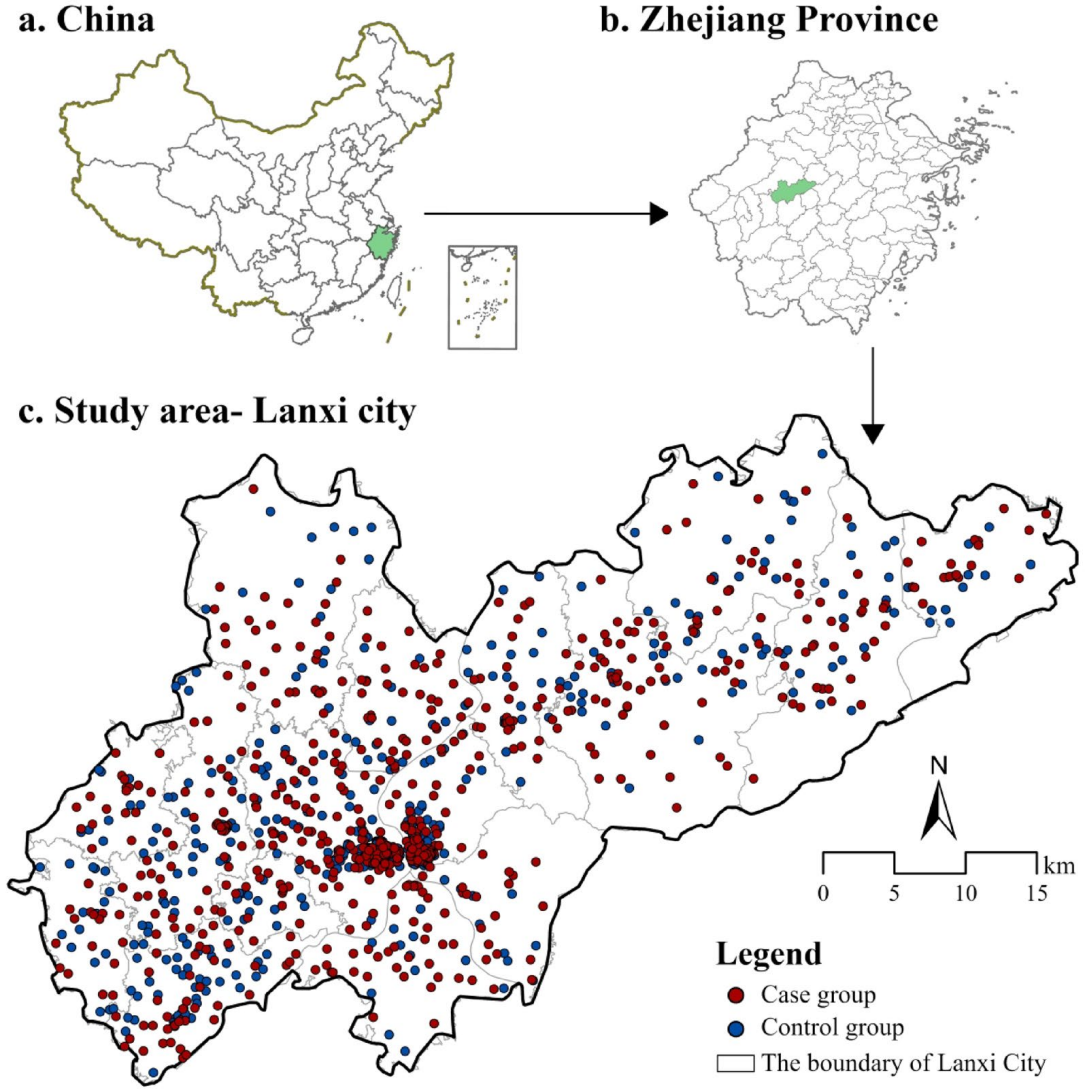
Study area and the locations of newly diagnosed PTB cases and matched controls

A case–control study was conducted in Lanxi, a city with a high incidence of PTB located in Zhejiang Province, China (Fig. 1). All PTB cases diagnosed between January 1, 2019, and December 31, 2021, were identified through the Web-based TB Information Management System (TBIMS) in China [24] and were based on the date of notification. Diagnoses and classifications of these cases followed the National Diagnostic Criteria for Pulmonary Tuberculosis (WS 288–2008 and WS 288–2017) and the Classification of Tuberculosis (WS 196–2017) published by the Ministry of Health of China. Patients with relapse diagnoses, missing address or variable data and recorded HIV status were excluded from our analyses. To establish the control group, individuals without PTB were randomly selected from the Lanxi Cloud-Based Healthcare Information System. By including both individuals who undergo passive physical examinations (e.g., routine employee health checkups) and those who actively seek health examinations, this system provides a representative sample with detailed health and exposure information suitable for the selection of non-PTB individuals. Controls were required to have physical examination records from the same year as their matched case and no documented HIV infection. Each case was matched with two controls based on sex and age (± 1 year). To minimize potential confounding caused by shared environmental exposures—such as similar levels of air pollution or living conditions—control subjects were limited to individuals who did not reside in the same street or town as the cases. This restriction helped reduce the likelihood of exposure misclassification due to geographic clustering. The residential addresses of all subjects were geocoded using the Amap geocoding service (https://lbs.amap.com/).

### Exposure assessment

In this study, we evaluated individual air pollutant exposure based on the average concentrations of air pollutants over the 24 months preceding either the diagnosis of PTB in case subjects or study entry for control subjects. The specific exposure window was chosen owing to the increased vulnerability to PTB and accelerated disease activation linked to air pollution during this period [5]. The PM_2.5_ [25, 26], ground-level O_3_ [27], and SO_2_ and NO_2_ [28, 29] data were obtained from the ChinaHighAirPollutants (CHAP) dataset (https://weijing-rs.github.io/product.html), which provides high-resolution measurements at 1 km for PM_2.5_, 1 km for O_3_. To ensure spatial resolution consistency across the entire 24-month exposure window (2017–2021), we used the 10-km resolution NO₂ and SO₂ data from the CHAP dataset, as 1-km products were only available from 2019 onward. The CHAP dataset is well regarded and has been widely used in public health research [30, 31]. For each participant, monthly exposure concentrations were extracted at the geographic centroid of their residential location, and the 24-month average exposure was calculated before the diagnosis date for cases or the physical examination date for controls.

Greenness exposure was estimated using the 16-days average Normalized Difference Vegetation Index (NDVI) at a resolution of 250 m. The NDVI data were obtained from the Terra MODIS, a satellite operated by the National Aeronautics and Space Administration. NDVI values ranged from 0 to 1, where higher positive values indicated greater vegetation coverage. In this study, we calculated the average NDVI over 24 months within buffer radii of 250 m, 500 m, and 1000 m around individual residence addresses following the same methodology used for assessing air pollution exposure.

### Covariate definition

Covariates—including demographic factors (sex and age), socioeconomic factors (education and working environment), individual behavioral characteristics (cigarette smoking, body mass index (BMI), and physical activity), household exposure factor (indoor air pollution), disease history (diabetes), and meteorological factors (temperature and precipitation)—were selected as potential confounding variables for the exposures (Table S1). Data for all covariates, except for the BMI, diabetes history, and meteorological factors, were gathered through face-to-face or telephone interviews using an electronic structured questionnaire (refer to Table S2 for detailed information). Indoor air pollution was assessed according to the cleanliness of the primary cooking fuel as a proxy, while BMI and diabetes history were obtained from physical examination records. Temperature and precipitation were obtained from the 1 km monthly mean temperature (10.11888/Meteoro.tpdc.270961) and precipitation dataset (10.5281/zenodo.3114194) for China [32].

### Statistical analysis

Two statistical methods were used in this study. First, a conditional logistic regression model was used to evaluate the independent relationship between air pollutants and PTB risks. Odds ratios (ORs) and 95% confidence intervals (CIs) were calculated for each one-unit increment in air pollutant levels. Second, the quantile-based g-computation (QgC) model was applied to estimate the overall effects (both additive and interactive effects) of the air pollutant mixture on PTB risks, as well as to determine the contribution of each pollutant within the mixture. The QgC model was chosen for its ability to estimate mixture effects while addressing multicollinearity and overfitting concerns inherent in traditional multi-pollutant models [33]. It combines exposures into a quantile-based index, providing stable and interpretable effect estimates even in the presence of correlated exposures. Additional, this approach allows for the allocation of beneficial or harmful weights to individual air pollutants within a mixture while also incorporating interaction terms to estimate their combined effects on PTB risks compared to weighted quantile sum (WQS) regression [34]. In this study, we estimated both the additive effects (i.e., without interaction terms) and interactive effects (i.e., with interaction terms) of air pollutants on PTB risks. The additive effects, defined as the cumulative impact of all individual pollutants, were assessed using the noboot function of the QgC model. The ORs for the additive effect of air pollutant mixture on PTB risks (i.e., mixture ORs) were calculated by increasing each air pollutant simultaneously by one quartile. Furthermore, to examine the interactive effects, we compared models that included interaction terms for pairwise pollutants with models that did not. The impact direction of the interactive effect for two individual air pollutants was inferred from the changes in mixture OR values between models with and without interaction terms. The fourth quantile of exposure variables was then chosen as the designated threshold in our models. Additionally, stratified analyses were further conducted by incorporating all covariates.

The moderating effect of greenness on the relationship between air pollutant mixtures and PTB risks was evaluated through a comparison between mixture ORs derived from models with and without interaction terms (air pollutant mixture $\times$ NDVI). A decrease in the mixture ORs upon including the interaction term indicates a negative moderating effect of greenness, suggesting that higher greenness reduces the correlation between mixed air pollutants and PTB risks. Conversely, an increase in the OR value denotes a positive moderating influence of greenness, indicating that higher greenness enhances the correlation.

Several sensitivity analyses were conducted to test the robustness of the results. First, we conducted subgroup analyses as a form of sensitivity analysis to examine whether the association between air pollutant mixtures and PTB risk varied by sociodemographic and behavioral factors. Z-tests were used to evaluate the statistical significance of between-group differences in the mixture effects. Second, to further examine the impact of selected confounding variables on the results, the models were re-evaluated with different sets of covariates. Third, the exposure data were also re-evaluated using a 12-month average period prior to PTB diagnosis or study enrollment, as the magnitude of the associations can vary depending on the exposure windows for greenness and air pollutant mixtures. Fourth, we included relapse PTB patients and their matched control subjects in the model to determine if their inclusion influenced the results. Fifth, to exclude the potential influence of the COVID-19 pandemic on our findings, we re-estimated the main multi-pollutant mixture model by restricting the dataset to the year 2019, prior to the outbreak in China.

All statistical analyses were conducted using R software 4.2.3.

## Results

### Descriptive analysis

**Fig. 2:**
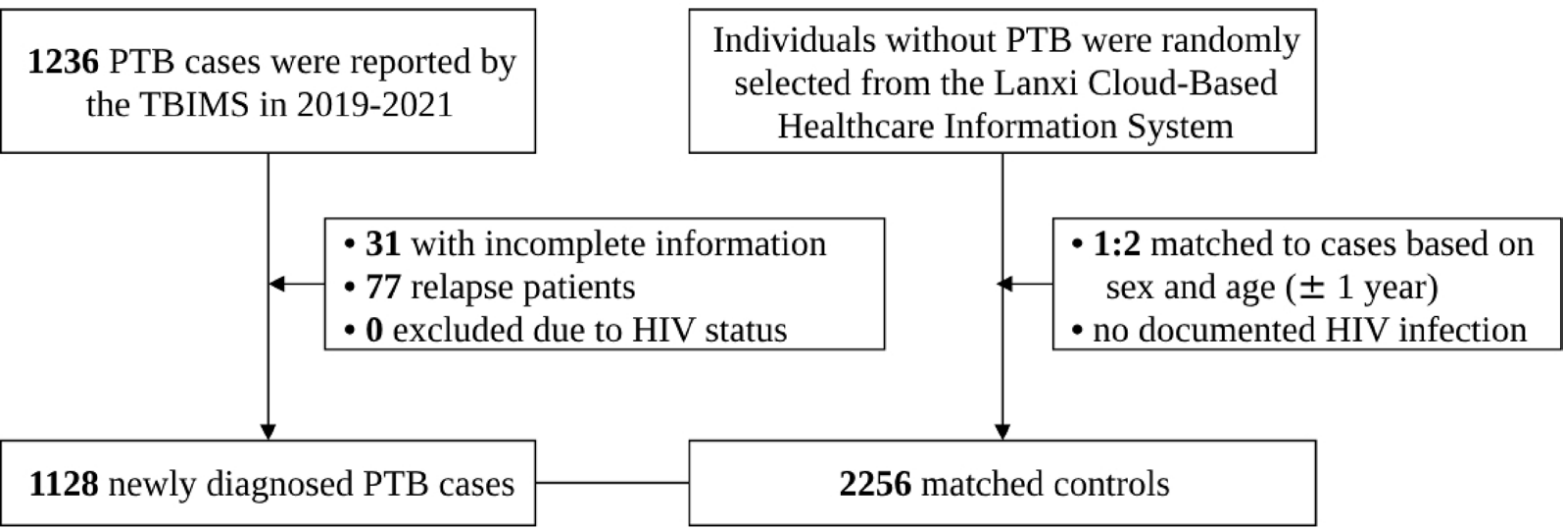
Flow diagram of cases and controls selection

During the study period from 2019 to 2021, 1236 PTB cases were reported by the TBIMS. After the exclusion of 31 cases with incomplete information and 77 relapse patients, our final analysis included 1128 PTB cases and 2256 controls (Fig. 2).

The characteristics of all subjects are presented in Table 1. Among the subjects included in the study, 2442 (72.16%) were male, and 1773 (52.39%) were older adults. PTB cases were associated with lower educational levels, more frequent engagement in outdoor occupations, higher exposure to indoor air pollution, and less participation in physical activities than the control group. PTB cases also had a higher prevalence of diabetes history, cigarette smoking, and suffering from malnutrition (BMI < 18.5) than the control group. Additionally, PTB cases exhibited a higher degree of exposure to elevated temperature and lower levels of precipitation than the control group.

**Table 1:** Characteristics of PTB cases and matched controls

Variables	All (n = 3384, %)	Cases (n = 1128, %)	Controls (n = 2256, %)
Sex			
Male	2442 (72.16%)	814 (72.16%)	1628 (72.16%)
Female	942 (27.84%)	314 (27.84%)	628 (27.84%)
Age			
≤ 18	71 (2.10%)	26 (2.31%)	45 (1.99%)
19–64	1540 (45.51%)	510 (45.21%)	1030 (45.66%)
≥ 65	1773 (52.39%)	592 (52.48%)	1181 (52.35%)
Education			
High school or below	3136 (92.67%)	1068 (94.68%)	2068 (91.67%)
College or above	248 (7.33%)	60 (5.32%)	188 (8.33%)
Working Environment			
Outdoor	2653 (78.40%)	960 (85.11%)	1693 (75.04%)
Indoor	731 (21.60%)	168 (14.89%)	563 (24.96%)
Indoor air pollution			
Low	3294 (97.34%)	1096 (97.16%)	2198 (97.43%)
High	90 (2.66%)	32 (2.84%)	58 (2.57%)
Cigarette smoking			
Never smoking	2427 (71.72%)	808 (71.63%)	1619 (71.76%)
Ever smoking	957 (28.28%)	320 (28.37%)	637 (28.24%)
Physical activity			
No	2890 (85.40%)	999 (88.56%)	1891 (83.82%)
Yes	494 (14.60%)	129 (11.44%)	365 (16.18%)
BMI			
< 18.5	251 (7.42%)	145 (12.85%)	106 (4.70%)
18.5 ≤ BMI < 25	2395 (70.77%)	864 (76.60%)	1531 (67.86%)
≥ 25	738 (21.81%)	119 (10.55%)	619 (27.44%)
History of diabetes			
No	3046 (90.01%)	996 (88.30%)	2050 (90.87%)
Yes	338 (9.99%)	132 (11.70%)	206 (9.13%)
Temperature (℃), mean ± std	18.78 ± 0.48	18.80 ± 0.48	18.77 ± 0.48
Precipitation (mm), mean ± std	1291.10 ± 7.12	1283.56 ± 7.29	1294.87 ± 6.70

std, standard deviation

As shown in Table 2, the 24-month average exposure to the four air pollutants in all subjects was lower than China’s guideline II levels. Specifically, the average concentrations of PM_2.5_, SO_2_, O_3_, and NO_2_ were 30.89 μg/m^3^, 8.82 μg/m^3^, 26.56 μg/m^3^, and 95.81 μg/m^3^, respectively. The estimated exposures for PM_2.5_, SO_2_, and NO_2_ were higher in the case group than in the control group. Conversely, individuals in the control group had higher O_3_ exposure than those in the case group. Additionally, subjects in the control group were more likely to reside in greener areas than those in the case group. In terms of spatial distribution, areas with higher annual average concentrations of PM_2.5_, NO_2_, SO_2_, and O_3_ were primarily located in the urban center of Lanxi (Fig. S1). According to the Spearman rank tests, the highest correlation was observed between the NDVI values within a 250 m buffer and a 500 m buffer (0.930, Table S3). Moreover, the correlation between PM_2.5_ and SO_2_ was relatively strong (0.828), while other pollutant pairs such as PM_2.5_ and NO_2_ (0.731) and NO_2_ and SO_2_ (0.563) showed moderate correlations. However, the incorporation of QgC in our analysis mitigates potential concerns regarding collinearity. Moreover, negative correlations were observed between O_3_ and the other pollutants, consistent with known atmospheric dynamics.

**Table 2:** Distribution of ambient air pollution concentrations and greenness across a 24-month period before the PTB diagnosis/entry into study for cases/matched controls

Exposures	All (n = 3384)			Cases (n = 1128)			Controls (n = 2256)		
	Mean ± std	Min	Max	Mean ± std	Min	Max	Mean ± std	Min	Max
PM_2.5_ (µg/m^3^)	30.89 ± 3.14	24.14	39.11	31.19 ± 3.20	24.20	39.11	30.75 ± 3.09	24.14	39.01
SO_2_ (µg/m^3^)	8.82 ± 1.31	7.28	12.40	8.89 ± 1.37	7.28	12.40	8.79 ± 1.28	7.36	12.40
NO_2_ (µg/m^3^)	26.56 ± 3.29	16.88	33.87	26.91 ± 3.11	18.29	33.79	26.39 ± 3.37	16.88	33.87
O_3_ (µg/m^3^)	95.81 ± 2.41	89.86	101.44	95.80 ± 2.38	90.06	101.44	95.83 ± 2.42	89.86	101.44
NDVI 250 m	0.43 ± 0.11	0.08	0.72	0.41 ± 0.10	0.12	0.68	0.43 ± 0.12	0.08	0.72
NDVI 500 m	0.46 ± 0.11	0.20	0.72	0.44 ± 0.11	0.20	0.71	0.46 ± 0.12	0.20	0.72
NDVI 1000 m	0.50 ± 0.10	0.27	0.77	0.49 ± 0.10	0.27	0.76	0.50 ± 0.10	0.27	0.77

NDVI 250 m, NDVI 500 m, and NDVI 1000 m represent average NDVI values within 250 m, 500 m, and 1000 m radius buffers around individual residence addresses, respectively

Min, minimum; Max, maximum; and std, standard deviation

### Association between individual air pollutants and PTB risks

**Fig. 3:**
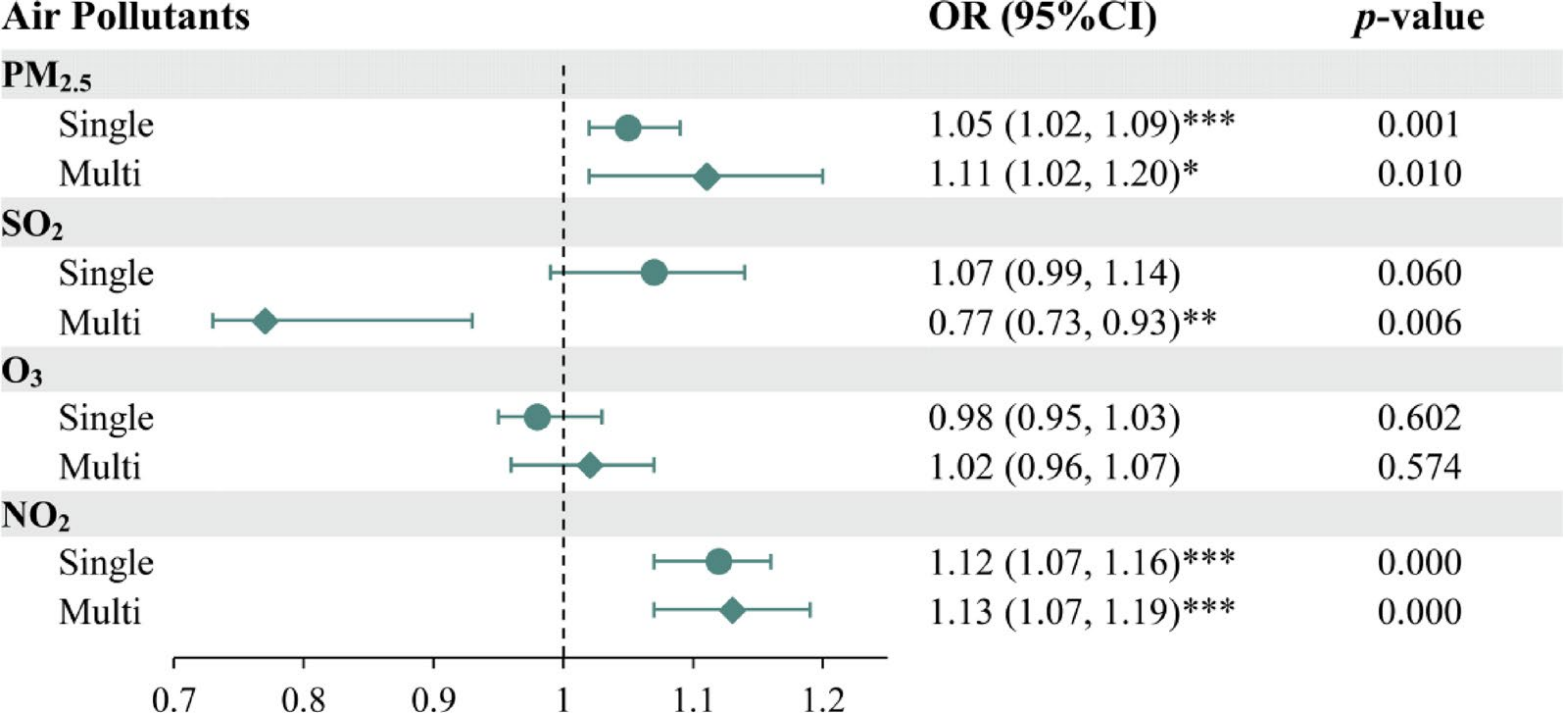
Conditional logistic regression estimated ORs and 95% CIs for the associations between individual air pollutants and PTB risks (single vs. multi). *Notes* Single-pollutant model, one pollutant (PM_2.5_ or SO_2_ or O_3_ or NO_2_) + matching factors and other covariates. Multi-pollutant model, PM_2.5_ + SO_2_ + O_3_ + NO_2_ + matching factors and other covariates. ***p < 0.001; **p < 0.01; and *p < 0.05.

The ORs and 95% CIs for the independent models are presented in Fig. 3. Both single-pollutant and multi-pollutant models were evaluated in this study, adjusting for matching factors and other covariates. In both single-pollutant and multi-pollutant models, significant positive associations were observed between PTB risks and exposure to PM_2.5_ and NO_2_. Additionally, in the multi-pollutant models, the effect estimate for SO_2_ shifted from indicating harm (OR: 1.07, 95% CI: 0.99, 1.14) to suggesting a protective effect (OR: 0.77, 95% CI: 0.73, 0.93) compared with the single-pollutant models. Notably, no significant association existed between O_3_ and PTB risks. The results remained generally consistent across models after adjusting for different sets of covariates (Table S5).

### Association between air pollutant mixture and PTB risks

The mixture OR and 95% CI were calculated to assess the additive effect of air pollutant mixtures on PTB risks. The analysis revealed that a one-quartile increase in exposure to the mixture of PM_2.5_, SO_2_, O_3_, and NO_2_ was significantly associated with an increased risk of PTB, with a mixture OR of 1.17 (95% CI: 1.07, 1.36; *p*-value: 0.043). Subgroup estimates for the pollutant mixture were generally consistent across demographic and behavioral categories. However, a significant difference was observed by physical activity level, with stronger associations among individuals engaging in higher-frequency physical activity (Table S4). No significant effect modification was found by sex, working environment, education, indoor air pollution, BMI, cigarette smoking and history of diabetes. Moreover, results from the sensitivity analysis using 2019 data alone showed similar effect estimates for the multi-pollutant mixture on PTB risk (Table S8).

**Fig. 4:**
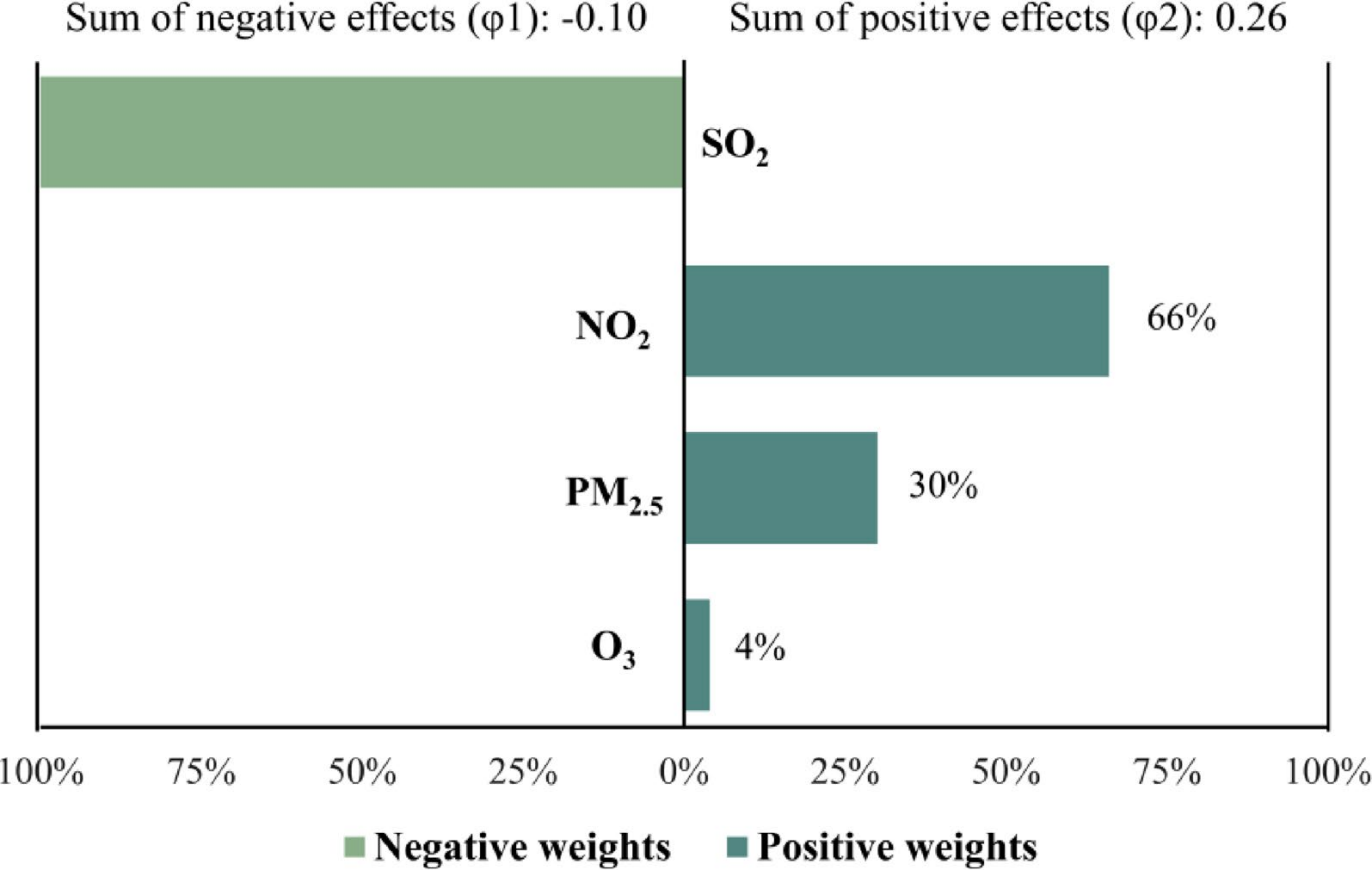
Proportion of the positive and negative effects of air pollutant mixture and PTB risks. *Notes* All models were adjusted for the covariates. The coefficient φ indicates the overall effect size of the combined exposures.

The coefficient *φ* indicates the overall effect size of the combined exposures. Among the pollutants in the mixture, NO_2_ had the largest positive contribution (66%), followed by PM_2.5_ (30%) and O_3_ (4%). In contrast, SO_2_ showed a negative contribution to the overall effect (Fig. 4). The overall effect of the predominantly positive contributions was 0.26, slightly offset by a minor negative effect of − 0.10. The results remained consistent when the mixture ORs were re-evaluated in models that included additional relapse PTB cases and considered different exposure windows (Table S6).

**Fig. 5:**
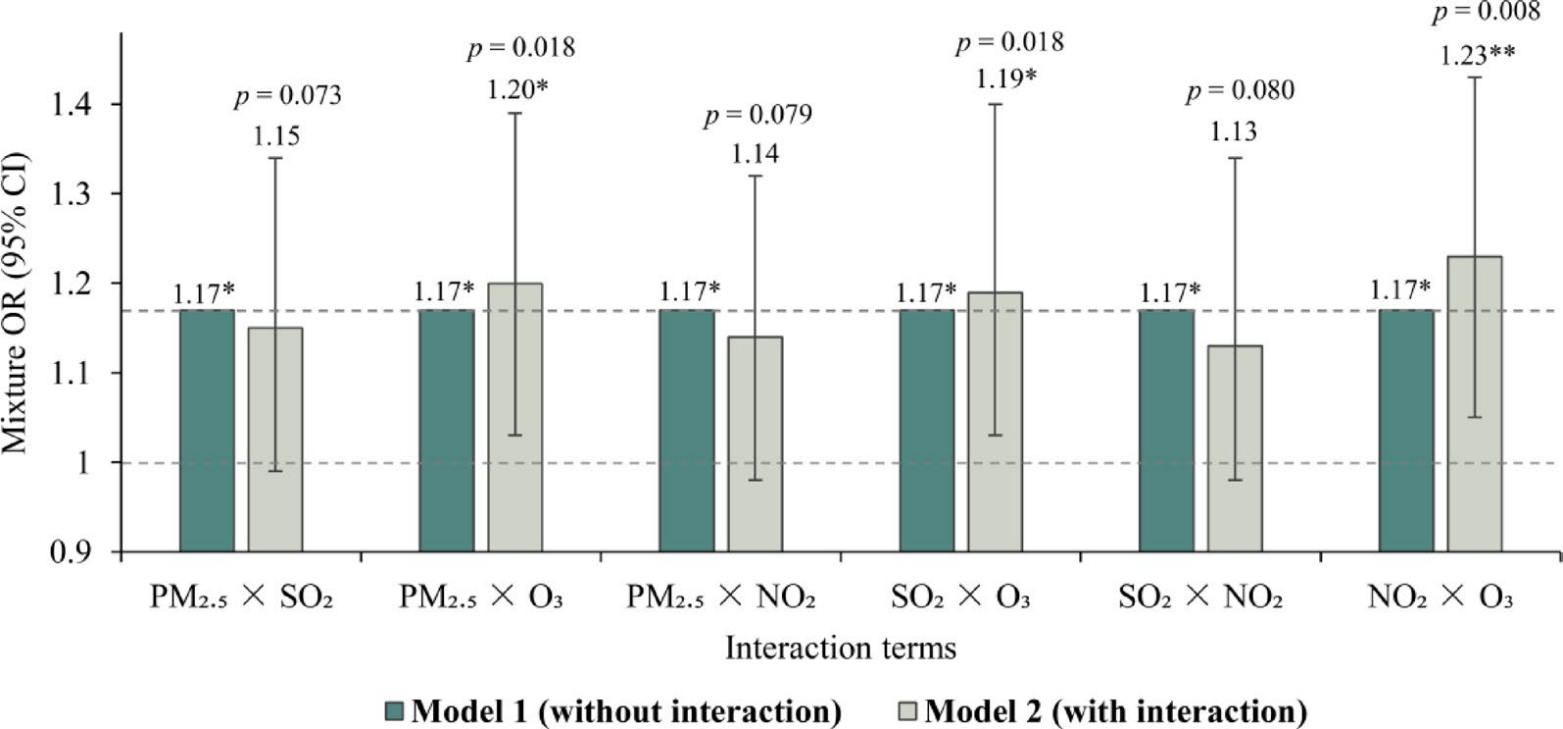
Potential interactive effect between air pollutant mixture and PTB risks. *Notes* ***p < 0.001; **p < 0.01; and *p < 0.05. All models were adjusted for the covariates. ^a^Model 1: the additive effect of air pollutant mixture on PTB risks; ^b^Model 2: introducing the interaction terms of any pairwise pollutants of PM_2.5_, SO_2_, O_3_ and NO_2_ based on Model 1. The directions of the interactive effect of air pollutant mixture on PTB risks were indicated by the change of the mixture ORs in Models 1 and 2. The increased mixture ORs in Model 2 compared with that of Model 1 represents the positive interactive effect of two air pollutants we introduced to PTB risks.

The interactive effect between individual air pollutants is presented in Table 3 and Fig. 5. When interaction terms between PM_2.5_ and O_3_, SO_2_ and O_3_, and NO_2_ and O_3_ were incorporated into Model 1 (in Table 3), the ORs significantly increased to 1.20 (95% CI 1.03, 1.39), 1.19 (95% CI 1.03, 1.40), and 1.23 (95% CI 1.05, 1.43) respectively. These results suggest that within the mixture of air pollutants, O_3_ may interact synergistically with any other pollutant, thereby increasing the risk of PTB beyond the additive effects of individual pollutants. When we used data from 2019 to exclude the potential influence of the COVID-19 pandemic, the interactive effects remained consistent with the main findings (Table S9).

**Table 3:** Potential interactive effect between air pollutant mixture and PTB risks

Interaction terms	Model 1^a^ (without interaction)		Model 2^b^ (with interaction)	
	Mixture OR (95% CI)	*p*-value	Mixture OR (95% CI)	*p*-value
PM_2.5_ $\times$ SO_2_	1.17 (1.07, 1.36)*	0.043	1.15 (0.99, 1.34)	0.073
PM_2.5_ $\times$ O_3_	1.17 (1.07, 1.36)*	0.043	1.20 (1.03, 1.39)*	0.018
PM_2.5_ $\times$ NO_2_	1.17 (1.07, 1.36)*	0.043	1.14 (0.98, 1.32)	0.079
SO_2_ $\times$ O_3_	1.17 (1.07, 1.36)*	0.043	1.19 (1.03, 1.40)*	0.018
SO_2_ $\times$ NO_2_	1.17 (1.07, 1.36)*	0.043	1.13 (0.98, 1.34)	0.080
NO_2_ $\times$ O_3_	1.17 (1.07, 1.36)*	0.043	1.23 (1.05, 1.43)**	0.008

All models were adjusted for the covariates. ^a^Model 1: the additive effect of air pollutant mixture on PTB risks; ^b^Model 2: introducing the interaction terms of any pairwise pollutants of PM_2.5_, SO_2_, O_3_, and NO_2_ based on Model 1. The directions of the interactive effect of air pollutant mixture on PTB risks were indicated by the change of the mixture ORs in Models 1 and 2. The increased mixture ORs in Model 2 compared with that of Model 1 represents the positive interactive effect of two air pollutants we introduced to PTB risks

***p < 0.001; **p < 0.01; and *p < 0.05

### Moderating effect of greenness on the association between air pollutant mixture and PTB risks

**Fig. 6:**
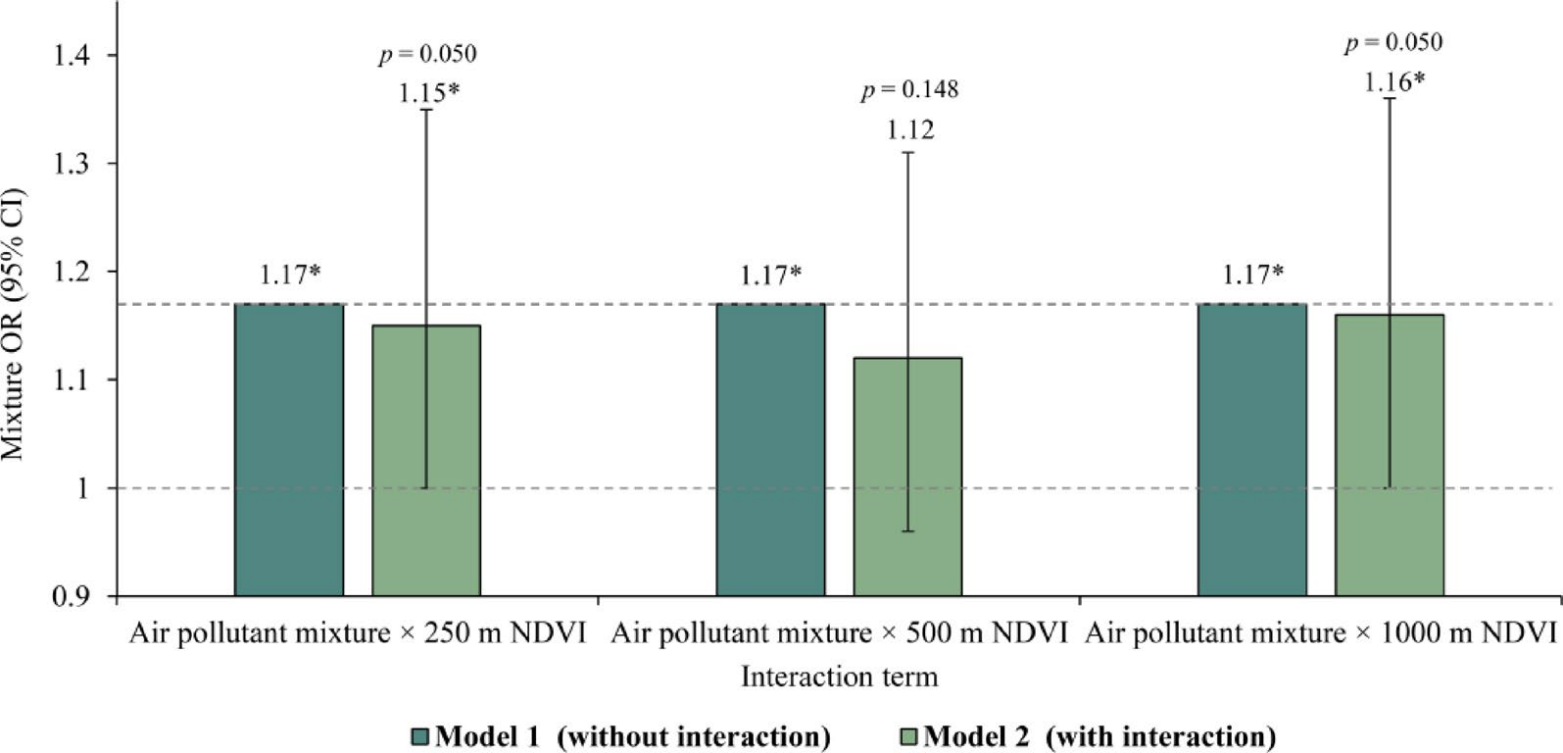
Examination of the moderating effect of greenness on the association between air pollutant mixture and PTB risks at three spatial scales. *Notes* ***p < 0.001; **p < 0.01; and *p < 0.05. All models were adjusted for the covariates. ^a^Model 1: the additive effect of air pollutant mixture on PTB risks. ^b^Model 2: introducing the interaction term of the NDVI and air pollutant mixture based on Model 1. The presence of a negative moderating effect of greenness on the correlation between mixed air pollutants and PTB risks is suggested by a reduction of the mixture ORs in Model 2 compared with Model 1. In contrast, an elevation in the mixture OR value denotes a positive moderating effect of greenness.

Table 4 and Fig. 6 present the mixture ORs with and without interaction terms between the NDVI and air pollutant mixture. Introducing an interaction term based on Model 1 at 250 m and 1000 m buffers led to a decrease in the ORs for mixed pollutants from 1.17 to 1.15 and 1.16, respectively (Model 2 in Table 4). These findings suggest that the NDVI serves as a moderating factor in the association between air pollutant mixtures and PTB risks at these two spatial scales. That is, areas with higher greenness levels exhibited a significantly lower PTB risk when exposed to a quarter-scale increase in air pollutant mixtures than areas with lower levels of greenness. Consistent results were also observed in an additional sensitivity analysis based on a 12-month exposure window (Table S7). However, no moderating effect of greenness was detected within the 500 m buffer.

**Table 4:** Examination of moderating effect of greenness on the association between air pollutant mixture and PTB risks at three spatial scales

Interaction terms	Model 1^a^ (without interaction)		Model 2^b^ (with interaction)	
	Mixture OR (95% CI)	*p*-value	Mixture OR (95% CI)	*p*-value
Air pollutant mixture $\times$ 250 m NDVI	1.17 (1.07, 1.36)*	0.043	1.15 (1.00, 1.35)*	0.050
Air pollutant mixture $\times$ 500 m NDVI	1.17 (1.07, 1.36)*	0.043	1.12 (0.96, 1.31)	0.148
Air pollutant mixture $\times$ 1000 m NDVI	1.17 (1.07, 1.36)*	0.043	1.16 (1.00, 1.36)*	0.050

All models were adjusted for the covariates. ^a^Model 1: the additive effect of air pollutant mixture on PTB risks. ^b^Model 2: introducing the interaction term of the NDVI and air pollutant mixture based on Model 1. The presence of a negative moderating effect of greenness on the correlation between mixed air pollutants and PTB risks is suggested by a reduction of the mixture ORs in Model 2 compared with Model 1. In contrast, an elevation in the mixture OR value denotes a positive moderating effect of greenness

***p < 0.001; **p < 0.01; and *p < 0.05.

## Discussion

### Independent effects of individual air pollutants on PTB risks

Our multi-pollutant model in Fig. 3 revealed that long-term exposure to SO_2_ was significantly negatively associated with PTB risk. Some studies have also reported similar findings [35, 36]; however, these studies mainly focused on short-term SO_2_ exposure. This research provides new evidence of an inverse relationship between long-term, low-level SO_2_ exposure and PTB risks, although the biological mechanisms behind this relationship remain unclear. The observed negative association could be attributed to the consistently low levels of SO_2_ in Lanxi, which are below China’s guideline I level (annual mean concentration of 20 µg/m^3^), and the infrequency of high-concentration exposure. These long-term exposures to lower levels of exogenous SO_2_ might induce oxidative damage to bacterial macromolecules, modulate cytokine responses (e.g., IL-6, TNF-α), and impair the survival or replication of *M.tb* by disrupting redox homeostasis and damaging alveolar macrophages [37–39].

### Combined effects of air pollutant mixture on PTB risks

Existing established evidence has shown positive associations between mixed pollutants exposure and various health outcomes, such as incident cardiovascular disease [9], cardiopulmonary mortality [8], and blood pressure [40]. Our results further extend this knowledge by revealing a consistent positive relationship between air pollutant mixtures and PTB risks in our additive models, which examined the cumulative effects of four pollutants. Regarding the contribution of each pollutant within the mixture, NO_2_, PM_2.5_, and O_3_ exhibited positive associations with PTB risks. Conversely, SO_2_ appeared to have a protective effect against PTB, but this effect was largely overshadowed by the impacts of the other three pollutants. These findings highlight that considering individual pollutants in isolation is not sufficient to represent the reality of human exposure to mixed pollutants because the potential protective effect of one pollutant may be offset by the adverse effects of others.

While PM_2.5_ is widely recognized as a major harmful pollutant [41, 42], our findings indicate that ambient NO_2_ had the most significant impact (66%) on the increased risk of PTB associated with pollutant mixtures, followed by PM_2.5_ (30%) and O_3_ (4%). This observation is consistent with a cross-sectional study conducted in northwestern China, which identified exposure to NO_2_ as the most significant environmental factor linked to tuberculosis (TB) [43]. Nevertheless, there is limited biological evidence that fails to provide substantial insight into the reasons for the contributions of each air pollutant. We speculate that the predominant pollutants identified may reflect major local sources of pollution in the study area. NO_2_ is generally regarded as an indicator of industrial and traffic-related pollutants. Given the significant presence of the cement and textile industries in Lanxi, individuals residing in this region are exposed to higher long-term levels of NO_2_, rendering them more vulnerable to the effects of NO_2_ than those of PM_2.5_.

In the interactive models, incorporating the interaction of various individual pollutants showed that the mixture OR exceeded the cumulative effects of individual pollutants. This implies that beyond their independent effects, interactions between pairs of pollutants within a mixed pollution scenario contributed further to PTB risks. Specifically, the interaction between O_3_ and any other air pollutant was found to significantly increase the risk of PTB. This synergistic harmful effect aligns with findings from recent epidemiological studies [44, 45]. For instance, a study conducted in Beijing, China, demonstrated that the co-exposure to PM_2.5_ and O_3_ had a synergistic effect on respiratory hospitalizations [45]. Similarly, another time-series study conducted in Beijing, China, found that exposure to O_3_ and NO_2_ simultaneously was positively associated with emergency room visits for respiratory diseases [44].

The harmful effects of the interaction between O_3_ and other air pollutants on PTB risks are biologically plausible. O_3_, a powerful oxidant, can exacerbate lung inflammation and oxidative stress caused by PM_2.5_ [46], potentially leading to the activation of *M.tb*. Moreover, NO_2_ is another major gaseous pollutant with strong oxidative capabilities that can contribute to cumulative oxidative stress on the human respiratory system when combined with O_3_ [44]. This interaction increases susceptibility to *M.tb* infection. Although population-based studies have identified a positive interaction between O_3_ and SO_2_ in relation to respiratory diseases [47], the underlying biological mechanisms remain unclear.

Our findings indicate that combinations involving O_3_ are likely a key factor contributing to additional PTB risks beyond what is predicted using the additive effects of individual pollutants. Although the positive effect and contribution of O_3_ to PTB risks were minimal in both the independent pollutant models and additive models, the harmful effect of O_3_ becomes critical when considering interactions with other pollutants. In China, the primary measures for reducing emissions have focused on controlling PM_2.5_ and NO_2_ and the implementation of strategies to reduce industrial pollution and vehicle exhaust emissions. However, considering the rising costs associated with addressing these pollutants independently and the synergistic harmful effects of O_3_ with other pollutants observed in this study, adopting a combined control approach for O_3_ and other pollutants could prove more cost-effective and beneficial for public health.

### Moderating effect of greenness

This study explored the moderating effect of greenness on the association between air pollutant mixture and PTB risks, addressing the limitations of studies that typically focus on single pollutants. We observed that the risks of PTB associated with a one-quarter increase in air pollutant mixtures were significantly lower in areas with higher levels of greenness than in areas with reduced levels of greenness. This result was consistent with previous studies that have identified a moderating effect of greenness on the relationship between single pollutants, such as PM_2.5_ and SO_2_, and TB risks [48, 49]. Our findings suggest that controlling air pollutant mixtures in areas with higher greenness is more effective in reducing the risk of PTB. Therefore, enhancing greenness should be regarded as an important public health strategy in addition to efforts aimed at reducing air pollution.

We propose three possible mechanisms that could explain this moderating effect of greenness within the framework of air pollutant mixture. First, individuals living in greener areas are often more engaged in outdoor activities [50, 51], which could enhance immune functions and help counteract the negative impacts of air pollutants on PTB risks [52]. Second, areas with abundant greenery may have a greater capacity for pollutant deposition, absorption, or enhanced dispersion, thereby reducing the ambient concentrations of individual particulates and gases [53, 54]. This may lead to a phenomenon in which regions with higher levels of greenness naturally experience lower levels of mixed air pollutants. Under such conditions, the likelihood of interactions and corresponding interaction intensity among pollutants may be diminished, which could, in turn, reduce susceptibility to *M.tb* infection. For instance, a reduction in particulate matter, such as PM_2.5_, can limit the entry of harmful gases such as O_3_ and NO_2_ into the lungs [13], thereby reducing the within-host activity of *M.tb.* Additionally, the cooling effects of greenness might reduce the photochemical reactions necessary for O_3_ formation [55, 56], thus reducing the oxidative interactions between O_3_ and other air pollutants and lessening their harmful effects on human lungs.

### Strengths and limitations

This study has several key strengths. First, the inclusion of detailed data on individual lifestyles and living environments provides significant advantages in mitigating the impact of unmeasured confounding factors and enhancing the statistical robustness of the study. Second, the use of the QgC model enables us to evaluate both the additive and interactive effects of air pollutants in mixtures. This approach helps mitigate potential biases in policy-making regarding air pollutant mixture control and prevents the implementation of inefficient health strategies owing to a lack of understanding of air pollutant interactions. Third, we examined the moderating effect of greenness on the association between mixed air pollutants and PTB risks, shifting the focus from individual pollutants to their combined effects and providing population-based evidence and theoretical insights into the interactions between greenness, air pollution, and health outcomes.

This study has several limitations as well. First, the inclusion of migrant cases might lead to biased results, although the relatively stable population in Lanxi mitigates this concern [57]. Second, as the findings are based on data from Lanxi—a region characterized by typically low air pollution levels—the findings may not be applicable to areas with higher pollution levels. Third, the potential for recall and self-reporting biases in the collection of individual data could lead to the misclassification of confounding variables; however, sensitivity analyses indicate that our results remain robust despite these potential biases. Fourth, due to data privacy restrictions, we could not construct alternative matched datasets with different case–control ratios for sensitivity analyses. While the control group was selected from a well-characterized, representative population with sufficient exposure and health data, the inability to examine other sampling strategies may limit the generalizability and robustness of our findings. Fifth, exposure assessment was based solely on participants’ residential addresses, which may not fully capture participants’ actual exposure in other locations such as workplaces or commuting environments. This simplified approach could lead to exposure misclassification, potentially biasing effect estimates and underestimating true associations. Sixth, the absence of other potential confounders such as household income and close contact history, may influence the observed associations. Future studies should incorporate these key variables to more precisely assess the associations between environmental exposures and PTB risks. Finally, although NDVI is a commonly used proxy for greenness, it does not account for individual-level accessibility or time spent in green environments, which may lead to exposure misclassification.

## Conclusions

This study introduced a joint analysis framework that focuses on the interactive effects among pollutants to evaluate the combined effects of ambient air pollutant mixtures on PTB risks, taking into account greenness as a moderating factor. Our findings indicated that long-term exposure to SO_2_ was negatively associated with PTB risk. Additionally, exposure to a mixture of air pollutants was positively associated with PTB risks, with NO_2_ making the most significant contribution to these heightened risks. While O_3_ had the lowest individual contribution, its interactive effects with other pollutants were strongly associated with higher PTB risks. Furthermore, the PTB risks associated with a one-quarter increase in air pollutant mixtures were significantly lower in areas with higher levels of greenness than in areas with lower levels of greenness. These findings highlight the importance of considering the moderating role of greenness in the context of air pollutant mixtures.

## Supplementary Information

Below is the link to the electronic supplementary material.

Supplementary Material 1.

## Data Availability

All data generated or analysed are included in this article and its supplementary information files. The corresponding authors can provide data upon reasonable request after completing all studies and sub-studies.
