# Supplementary material for: Beyond additive effects: examining the combined impact of air pollutant interactions on pulmonary tuberculosis in China
Source: BMC Public Health. 2025 Sep 24;25:3102. doi: 10.1186/s12889-025-24421-5 (PMC12462355; doi:10.1186/s12889-025-24421-5)
Supplement: Supplementary file 1 — Supplementary Material 1. [file 12889_2025_24421_MOESM1_ESM.docx]

# Supplementary Materials

**Table S1.** Description and data sources for covariates.

**Table S2.** Structured electronic questionnaire designed for face-to-face or telephone interviews.

**Table S3.** Spearman correlations among air pollutants and greenness exposure.

**Table S4.** Additive effects of air pollutant mixture on PTB risks across subgroups.

**Table S5.** Re-estimated additive effects of air pollutant mixture on PTB risks after adjusting for different sets of covariates.

**Table S6.** Re-estimated additive effects of air pollutant mixture on PTB risks including the relapse PTB patients and matched control subjects as well as considering changes in the exposure window.

**Table S7.** Moderating effects of greenness on the association between air pollutant mixture and PTB risks using the 12-month average values.

**Table S8.** Re-estimated additive effects of air pollutant mixture on PTB risks using data from the year of 2019.

**Table S9.** Potential interactive effect between air pollutant mixture and PTB risks using data from the year of 2019.

**Fig. S1.** Spatial distribution of annual average concentrations of PM₂.₅, NO_2_, O₃ and SO_2_ in Lanxi City during the study period.

**Table S1**

Description and data sources for covariates.

| Covariates | Description | Data sources |
| --- | --- | --- |
| **Sex** |  |  |
| Male | - |  |
| Female | - |  |
| **Age** |  |  |
| Children | ≤ 18 |  |
| Young and middle-aged | 19-64 |  |
| Elderly | ≥ 65 |  |
| **Education** |  |  |
| High school or below | No formal education (illiterate); Elementary school; Middle school; High school; Vocational school |  |
| College or above | Two/Three Year College/Associate degree; Four Year College/Bachelor’s degree; Graduate degree |  |
| **Working Environment** |  |  |
| Outdoor | Agricultural, forestry, husbandry and fishery producers; Production and transportation workers |  |
| Indoor | Managers; Professionals and technicians; Clerks; Commercial and service workers |  |
| **Indoor air pollution** |  |  |
| Low | Cooking using liquefied petroleum gas, natural gas and electric |  |
| High | Cooking using coal and wood burning; |  |
| **Cigarette smoking** |  |  |
| Never smoking | Never smoking |  |
| Ever smoking | Smoking and quit smoking |  |
| **Physical activity** |  |  |
| No | Never |  |
| Yes | Almost daily; At least once a week; Occasionally |  |
| **BMI** |  |  |
| Malnutrition | ＜ 18.5 | Retrieved from physical examination record |
| Normal weight | 18.5 ≤ BMI ＜ 25 |  |
| Overweight | ≥ 25 |  |
| **History of diabetes** |  |  |
| No | - | Diabetes was ascertained either through a documented prior diagnosis or by means of a positive fasting glucose test |
| Yes | - |  |
| **Temperature** | - | The National Meteorological Data Center in China (http://data.cma.cn/site) |
| **Precipitation** | - |  |

**Table S2**

Structured electronic questionnaire designed for face-to-face or telephone interviews.

| **Questions** | **Options** |
| --- | --- |
| **Education** |  |
| What is the highest level of education you completed? | **🗆** No formal education (illiterate) **🗆** Elementary school  **🗆** Middle school **🗆** High school **🗆** Vocational school  **🗆** Two/Three Year College / Associate degree  **🗆** Four Year College / Bachelor’s degree  **🗆** Graduate degree **🗆** Other |
| **Occupation** |  |
| What is your current occupation | **🗆** Managers **🗆** Professionals and technicians  **🗆** Clerks **🗆** Commercial and service workers  **🗆** Agricultural, forestry, husbandry and fishery producers  **🗆** Production and transportation workers  **🗆** Can’t be specified **🗆** No works |
| **Cooking fuel** |  |
| What is the main source of cooking fuel? | **🗆** Liquefied petroleum gas **🗆** Coal **🗆** Natural gas  **🗆** Wood burning **🗆** Electric **🗆** Other |
| **Smoking** |  |
| Whether you have a habit of smoking? | **🗆** Smoking **🗆** Never smoking **🗆** Quit smoking |
| **Physical** **activity** |  |
| The frequency of physical exercise | **🗆** Almost daily **🗆** At least once a week  **🗆** Occasionally **🗆** Never |

**Table S3**

Spearman correlations among air pollutants and greenness exposure.

| Exposures | Correlation coefficient | | | | | | |
| --- | --- | --- | --- | --- | --- | --- | --- |
|  | PM_2.5_ | SO_2_ | NO_2_ | O_3_ | NDVI 250 m | NDVI 500 m | NDVI 1000 m |
| PM_2.5_ | 1.000 | 0.828* | 0.731* | -0.389* | -0.354* | -0.385* | -0.404* |
| SO_2_ |  | 1.000 | 0.563* | -0.573* | -0.131* | -0.128* | -0.141* |
| NO_2_ |  |  | 1.000 | -0.086* | -0.657* | -0.709* | -0.705* |
| O_3_ |  |  |  | 1.000 | -0.120* | -0.166* | -0.176* |
| NDVI 250 m |  |  |  |  | 1.000 | 0.930* | 0.858* |
| NDVI 500 m |  |  |  |  |  | 1.000 | 0.917* |
| NDVI 1000 m |  |  |  |  |  |  | 1.000 |

Notes: NDVI 250 m & NDVI 500 m & NDVI 1000 m, average NDVI values within 250 m, 500 m and 1000 m radius buffers. *** *p* ＜ 0.001; ** *p* ＜ 0.01; * *p* ＜0.05.

**Table S4**

Additive effects of air pollutant mixture on PTB risks across subgroups.

| Subgroups | Mixture OR（95% CI） | Z-test *p*-value |
| --- | --- | --- |
| **Sex** |  |  |
| Male | 1.47 (0.96, 2.23) |  |
| Female | 1.56 (0.81, 3.02) | 0.88 |
| **Working environment** |  |  |
| Indoor | 1.38 (0.90, 2.12) |  |
| Outdoor | 1.17 (0.99, 1.39) | 0.48 |
| **Education** |  |  |
| High school or below | 0.86 (0.39, 1.89) |  |
| College or above | 1.21 (1.04, 1.43)** | 0.41 |
| **Indoor air pollution** |  |  |
| Low | 1.16 (0.99, 1.36) |  |
| High | 1.91 (0.52, 2.66) | 0.24 |
| **BMI** |  |  |
| 18.5 ≤ BMI ＜ 25 | 1.07 (0.89, 1.27) |  |
| ＜ 18.5 | 1.41 (0.83, 2.42) | 0.34 |
| ≥ 25 | 1.39 (0.89, 2.18) | 0.97 |
| **Cigarette smoking** |  |  |
| Never smoking | 1.14 (0.95, 1.38) |  |
| Ever smoking | 1.22 (0.91, 1.65) | 0.71 |
| **Physical activity** |  |  |
| Low frequency | 1.11 (0.94, 1.31) |  |
| High frequency | 1.84 (1.02, 1.22)* | 0.00 |
| **History of diabetes** |  |  |
| No | 1.13 (0.96, 1.34) |  |
| Yes | 1.66 (1.02, 2.71)* | 0.14 |

Notes: *** *p* ＜ 0.001; ** *p* ＜ 0.01; * *p* ＜ 0.05.

**Table S5**

Re-estimated additive effects of air pollutant mixture on PTB risks after adjusting for different sets of covariates.

| Sensitive analysis models | Mixture OR（95% CI） | *p*-value |
| --- | --- | --- |
| Model 1 ^a^ | 1.13 (0.99, 1.29) | 0.06 |
| Model 2 ^b^ | 1.18 (1.03, 1.34)* | 0.02 |
| Model 3 ^c^ | 1.19 (1.04, 1.37)** | 0.01 |
| Model 4 ^d^ | 1.17 (1.07, 1.36)* | 0.04 |

Notes: ^a^ adjusting for age and sex; ^b^ adjusting for age, sex and socioeconomic factors; ^c^ adjusting for age, sex, socioeconomic factors, individual behavioral factors and history of disease; ^d^ adjusting for all covariates. *** *p* ＜ 0.001; ** *p* ＜ 0.01; * *p* ＜ 0.05.

**Table S****6**

Re-estimated additive effects of air pollutant mixture on PTB risks including the relapse PTB patients and matched control subjects as well as considering changes in the exposure window.

| Sensitivity analysis | Mixture OR（95% CI） | *p*-value |
| --- | --- | --- |
| New diagnosed PTB cases + relapse PTB cases | 1.22 (1.05, 1.43)** | 0.01 |
| Exposure window of 12 months | 1.35 (1.18, 1.56)*** | 0.00 |

Notes: *** *p* ＜ 0.001; ** *p* ＜ 0.01; * *p* ＜ 0.05.

**Table S7**

Moderating effects of greenness on the association between air pollutant mixture and PTB risks using the 12-month average values.

| Interaction terms | Mixture OR (95% CI) | |
| --- | --- | --- |
|  | Model 1 ^a^ (Without interaction) | Model 2 ^b^ (With interaction) |
| Air pollutant mixture $\times$ 250 m NDVI | 1.35 (1.18, 1.56)*** | 1.25 (1.08, 1.44)*** |
| Air pollutant mixture $\times$ 500 m NDVI | 1.35 (1.18, 1.56)*** | 1.24 (1.07, 1.42)*** |
| Air pollutant mixture $\times$ 1000 m NDVI | 1.35 (1.18, 1.56)*** | 1.30 (1.15, 1.51)*** |

Notes: NDVI 250 m & NDVI 500 m & NDVI 1000 m, average NDVI values within 250 m, 500 m and 1000 m radius buffers around individual residence addresses, respectively. ^a^ Model 1: OR of air pollutant mixture on PTB risks after adjusting for the matching factors and all covariates. ^b^ Model 2: introducing the interaction term of NDVI and air pollutant mixture based on Model 1. *** *p* ＜ 0.001; ** *p* ＜ 0.01; * *p* ＜ 0.05.

**Table S8**

Re-estimated additive effects of air pollutant mixture on PTB risks using data from the year of 2019.

| Sensitivity analysis | Mixture OR（95% CI） | *p*-value |
| --- | --- | --- |
| PTB cases and controls from 2019 | 2.09 (1.24, 5.01)*** | 0.00 |

Notes: *** *p* ＜ 0.001; ** *p* ＜ 0.01; * *p* ＜ 0.05.

**Table S9**

Potential interactive effect between air pollutant mixture and PTB risks using data from the year of 2019.

| Interaction terms | Model 1 ^a^ (without interaction) | | Model 2 ^b^ (with interaction) | |
| --- | --- | --- | --- | --- |
|  | Mixture OR (95% CI) | *p*-value | Mixture OR (95% CI) | *p*-value |
| PM_2.5_ $\times$ SO_2_ | 2.09 (1.24, 5.01)*** | 0.000 | 1.23 (0.82, 2.18) | 0.408 |
| PM_2.5_ $\times$ O_3_ | 2.09 (1.24, 5.01)*** | 0.000 | 1.38 (1.22, 2.69)*** | 0.000 |
| PM_2.5_ $\times$ NO_2_ | 2.09 (1.24, 5.01)*** | 0.000 | 1.34 (0.81, 2.34) | 0.280 |
| SO_2_ $\times$ O_3_ | 2.09 (1.24, 5.01)*** | 0.000 | 1.48 (1.23, 2.81)*** | 0.000 |
| SO_2_ $\times$ NO_2_ | 2.09 (1.24, 5.01)*** | 0.000 | 1.09 (0.83, 1.95) | 0.692 |
| NO_2_ $\times$ O_3_ | 2.09 (1.24, 5.01)*** | 0.000 | 1.17 (1.22, 2.88)*** | 0.000 |

Notes: *** *p* < 0.001; ** *p* < 0.01; and * *p* < 0.05. All models were adjusted for the covariates. ^a^ Model 1: the additive effect of air pollutant mixture on PTB risks; ^b^ Model 2: introducing the interaction terms of any pairwise pollutants of PM_2.5_, SO_2_, O_3_, and NO_2_ based on Model 1. The directions of the interactive effect of air pollutant mixture on PTB risks were indicated by the change of the mixture ORs in Models 1 and 2. The increased mixture ORs in Model 2 compared with that of Model 1 represents the positive interactive effect of two air pollutants we introduced to PTB risks.


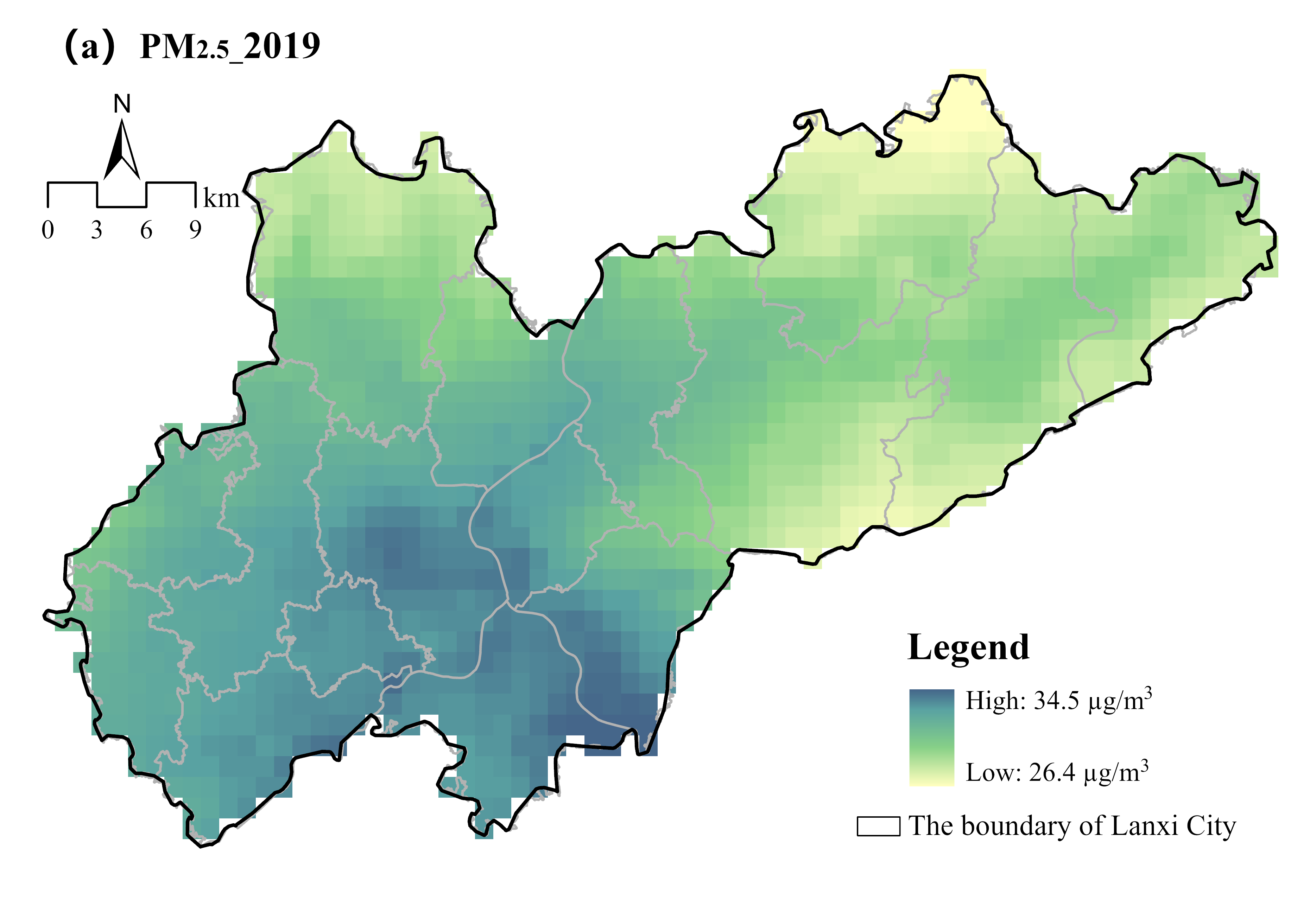

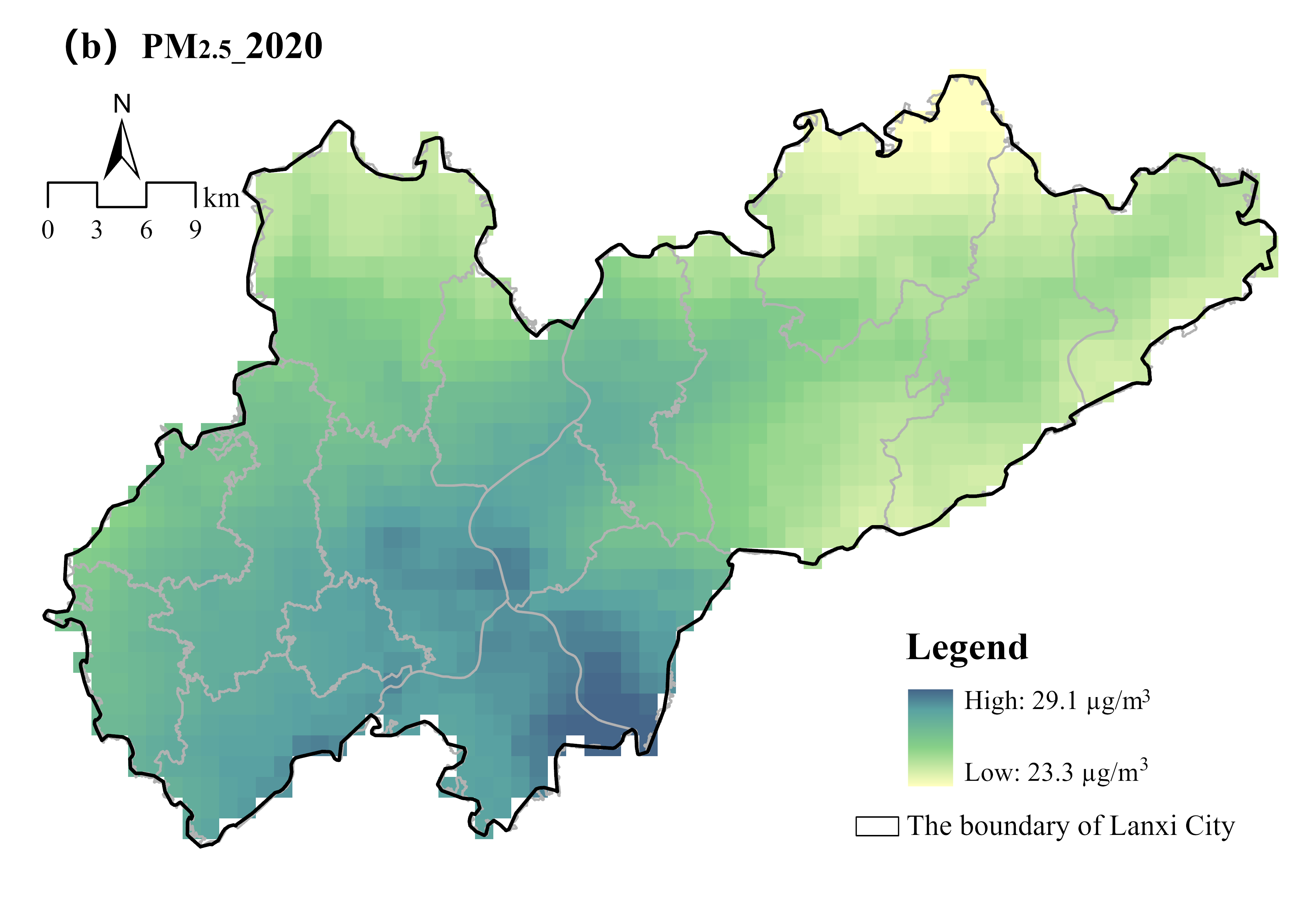

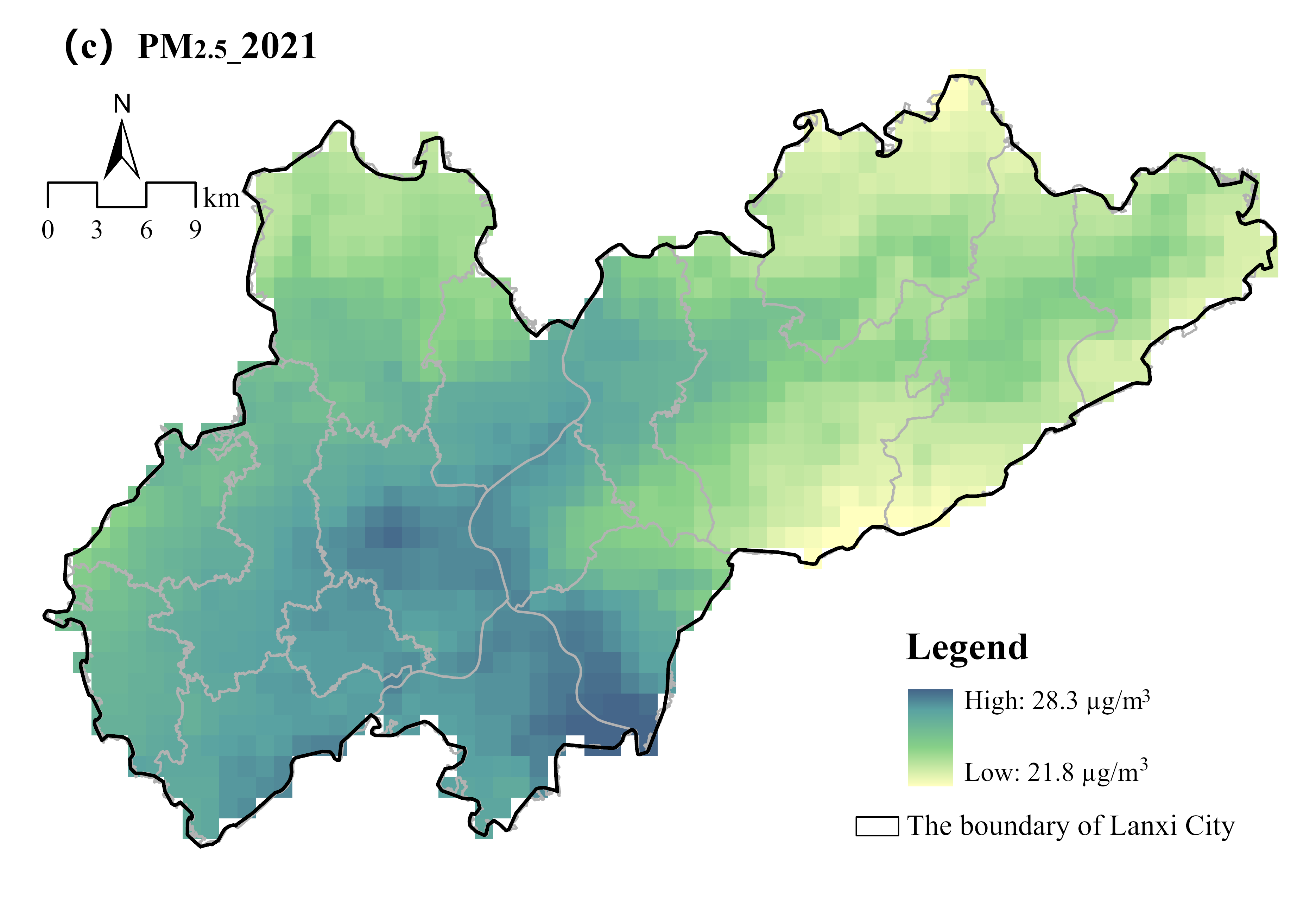


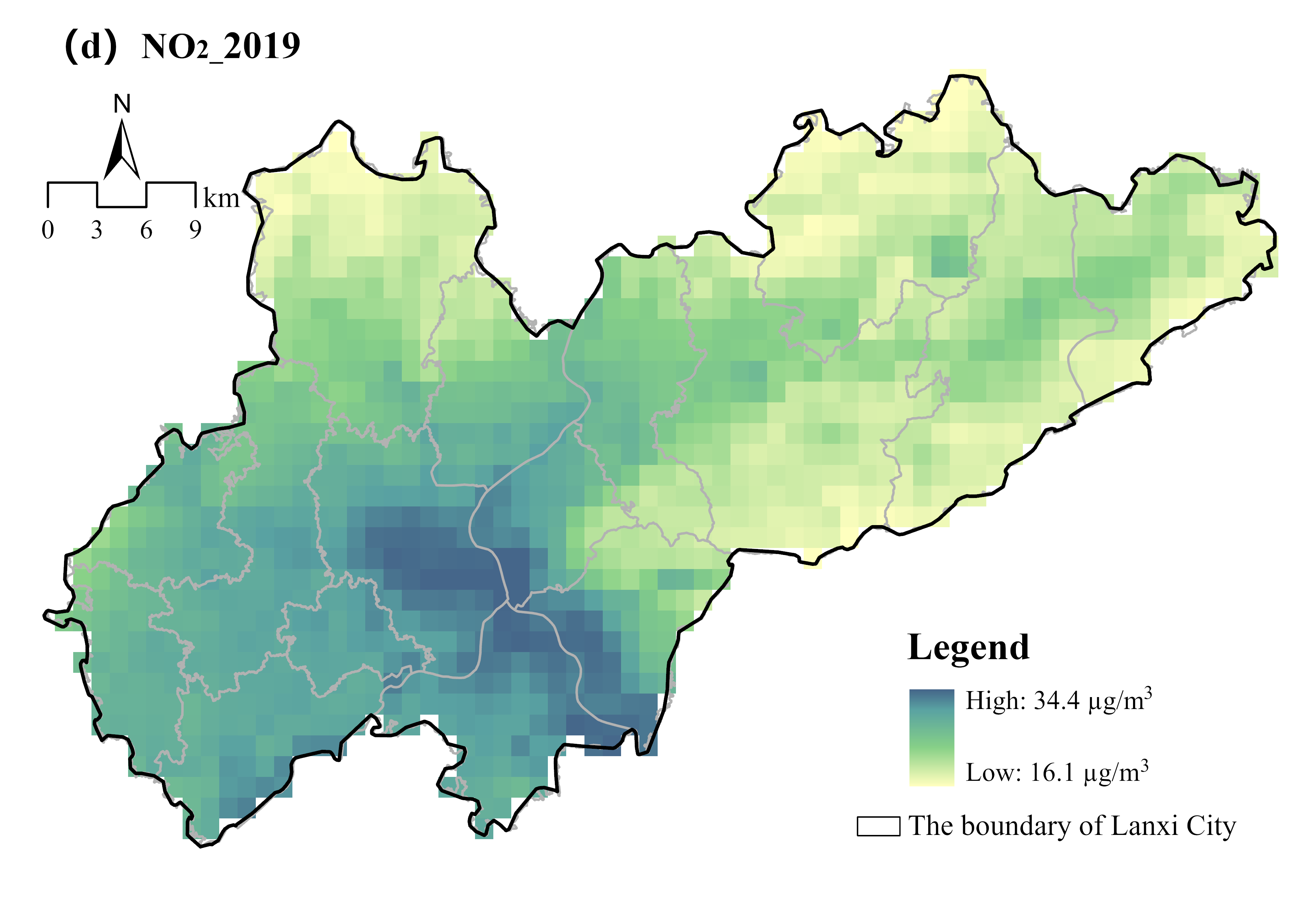

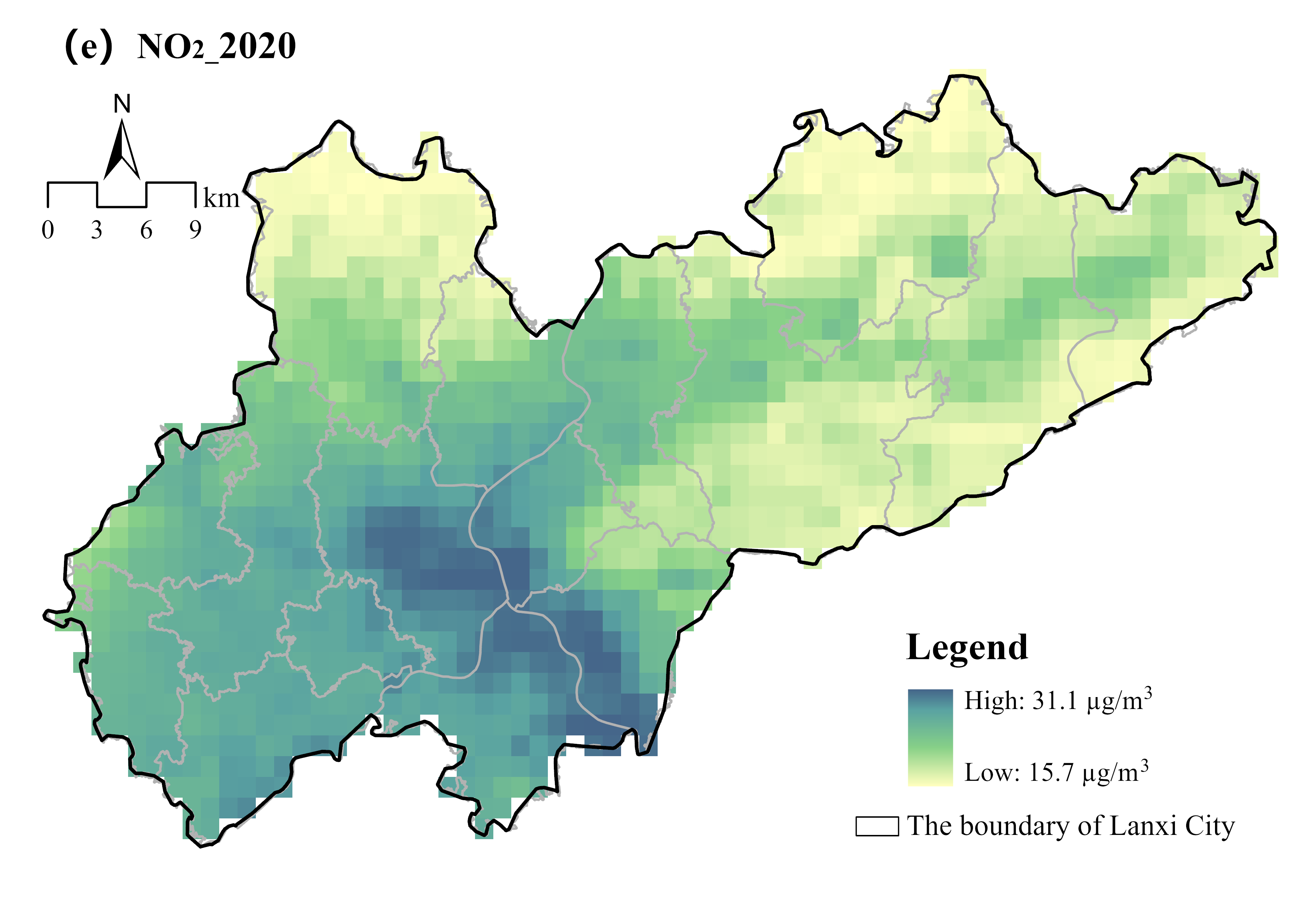

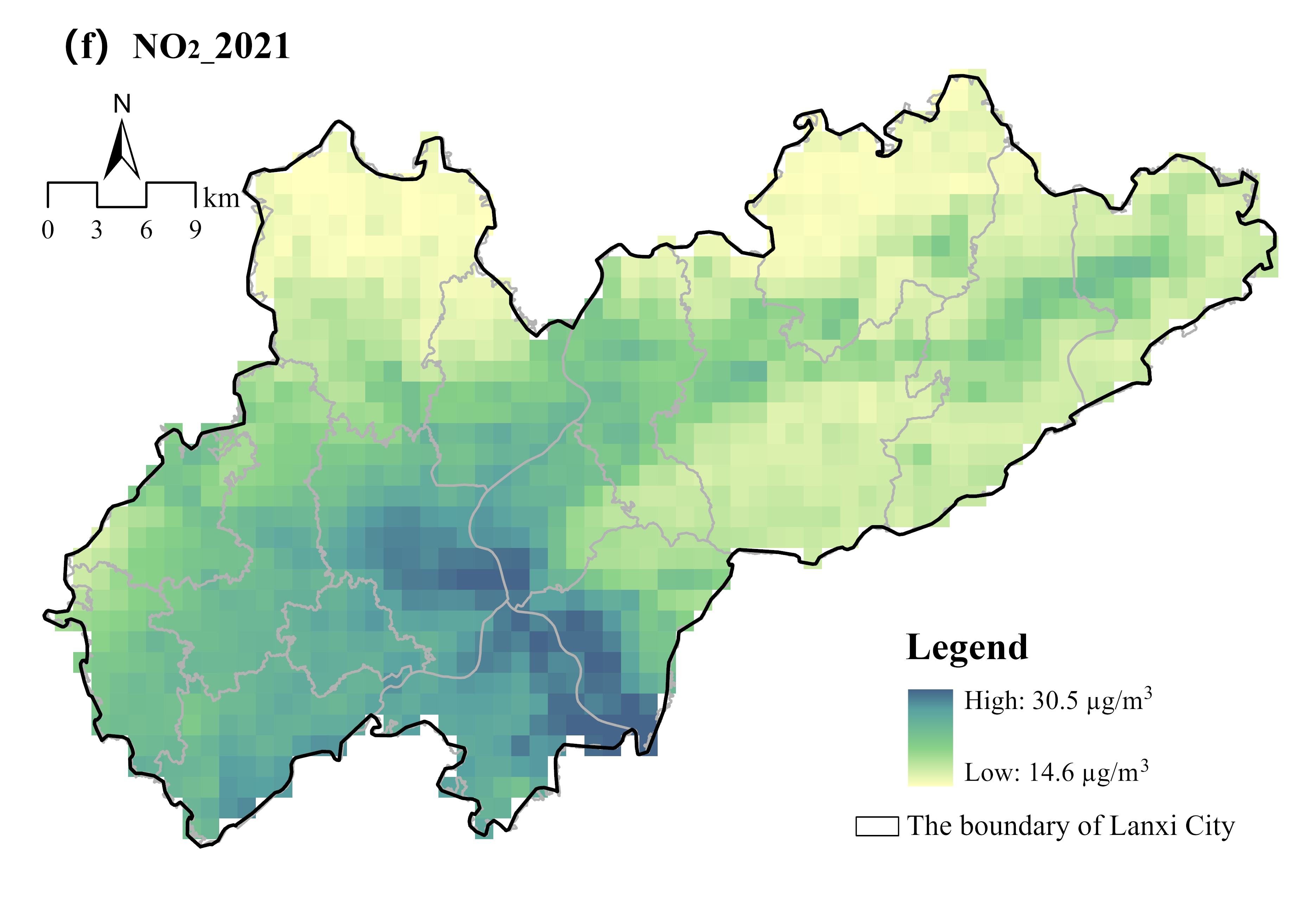


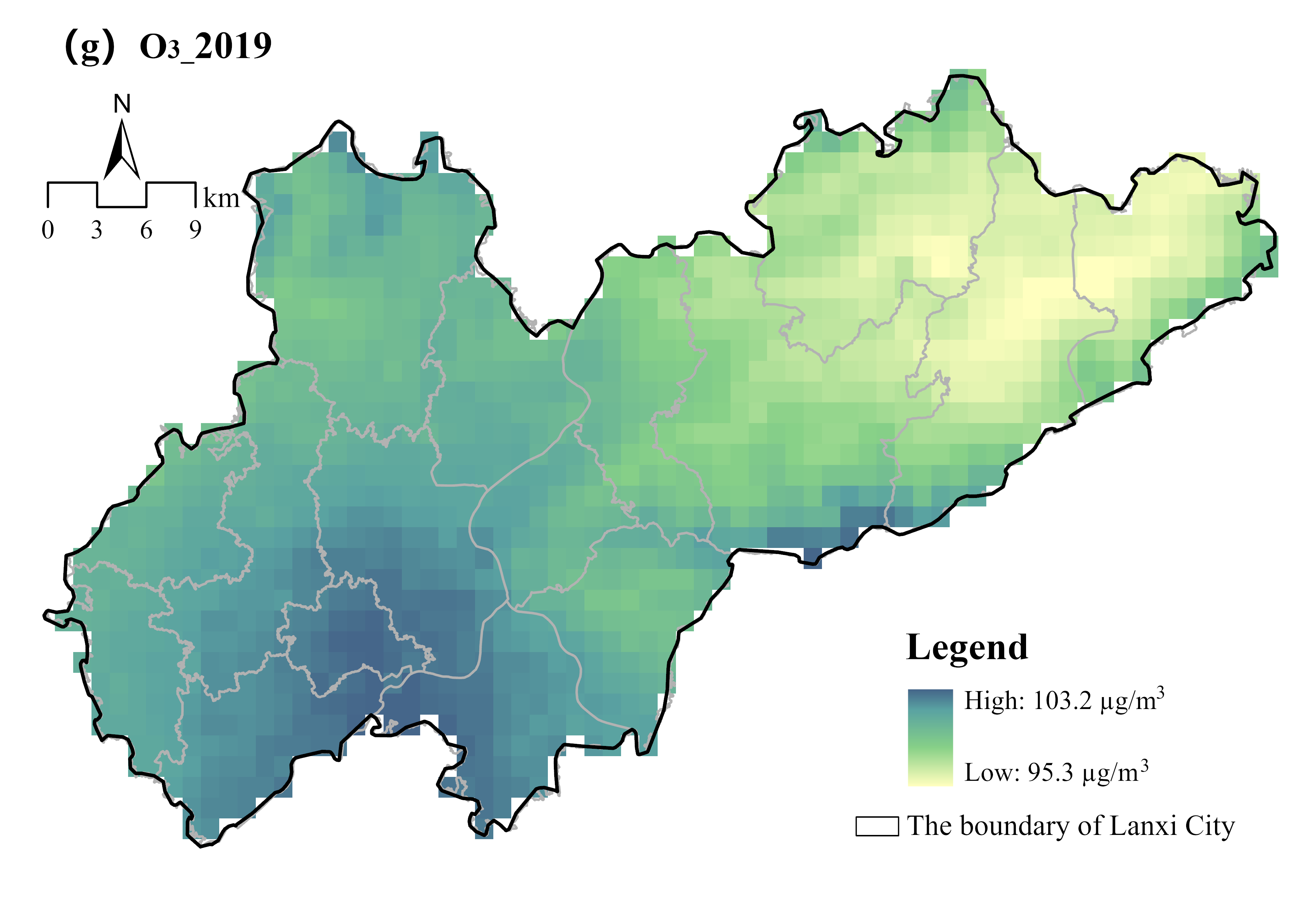

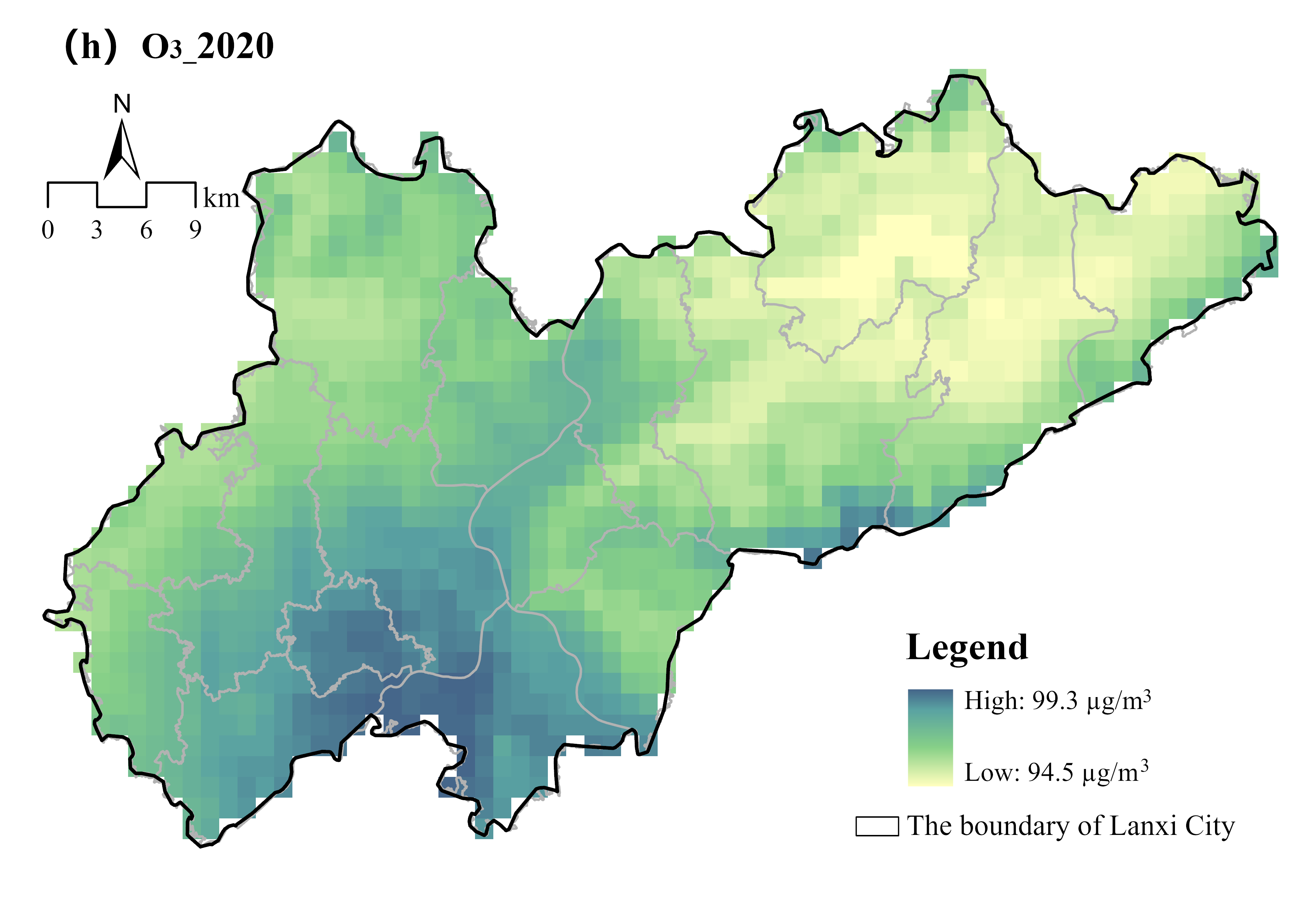

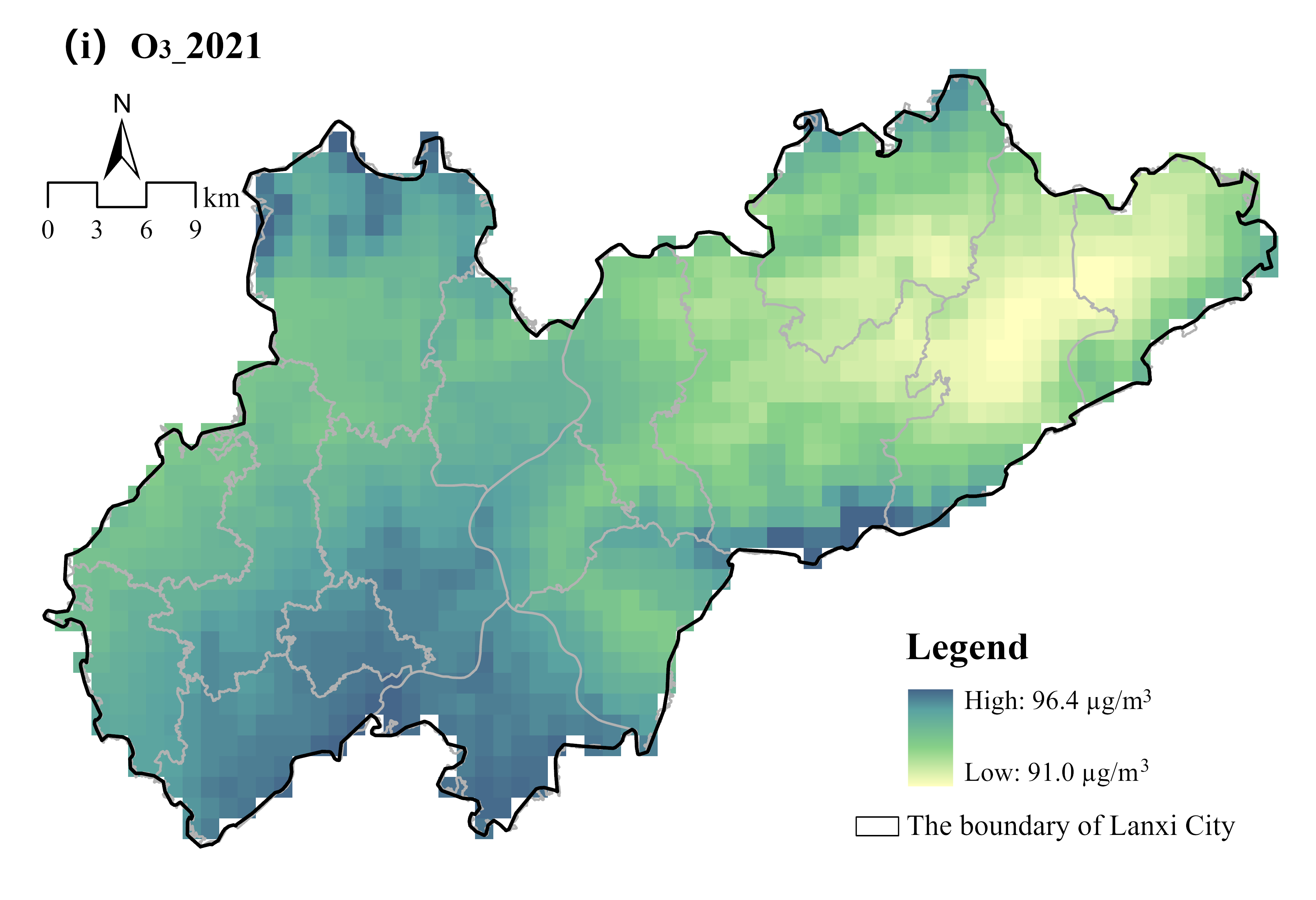


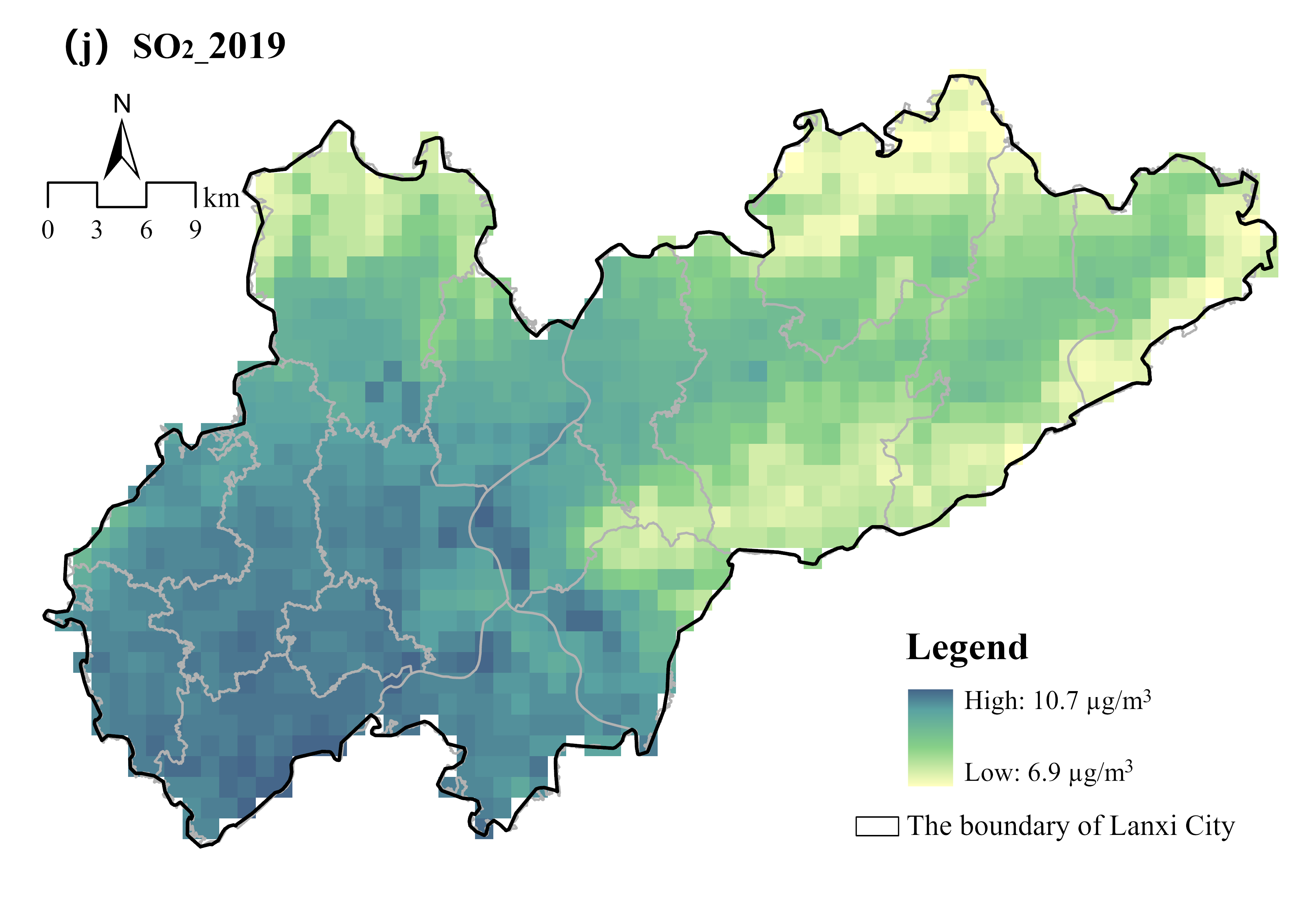

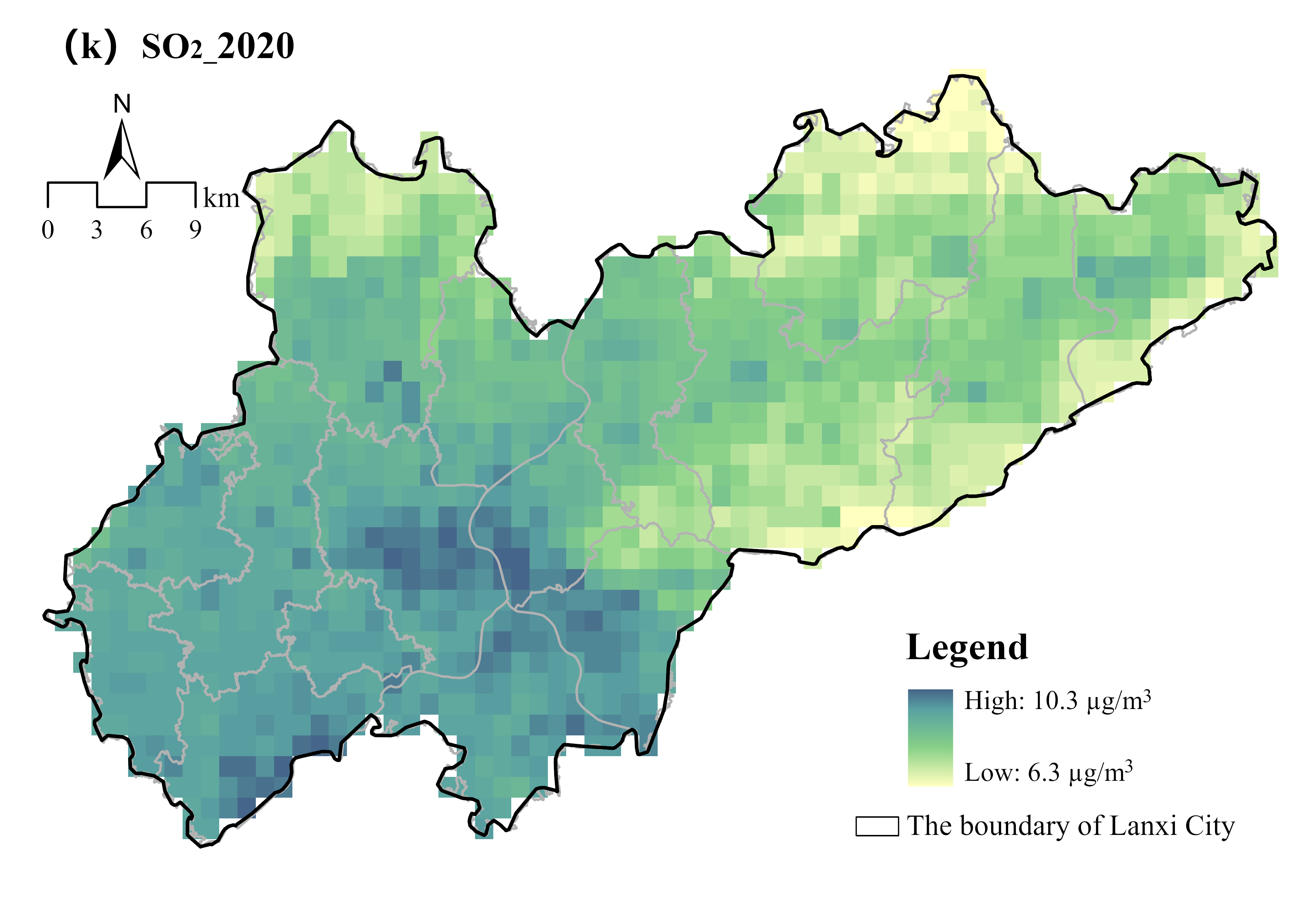

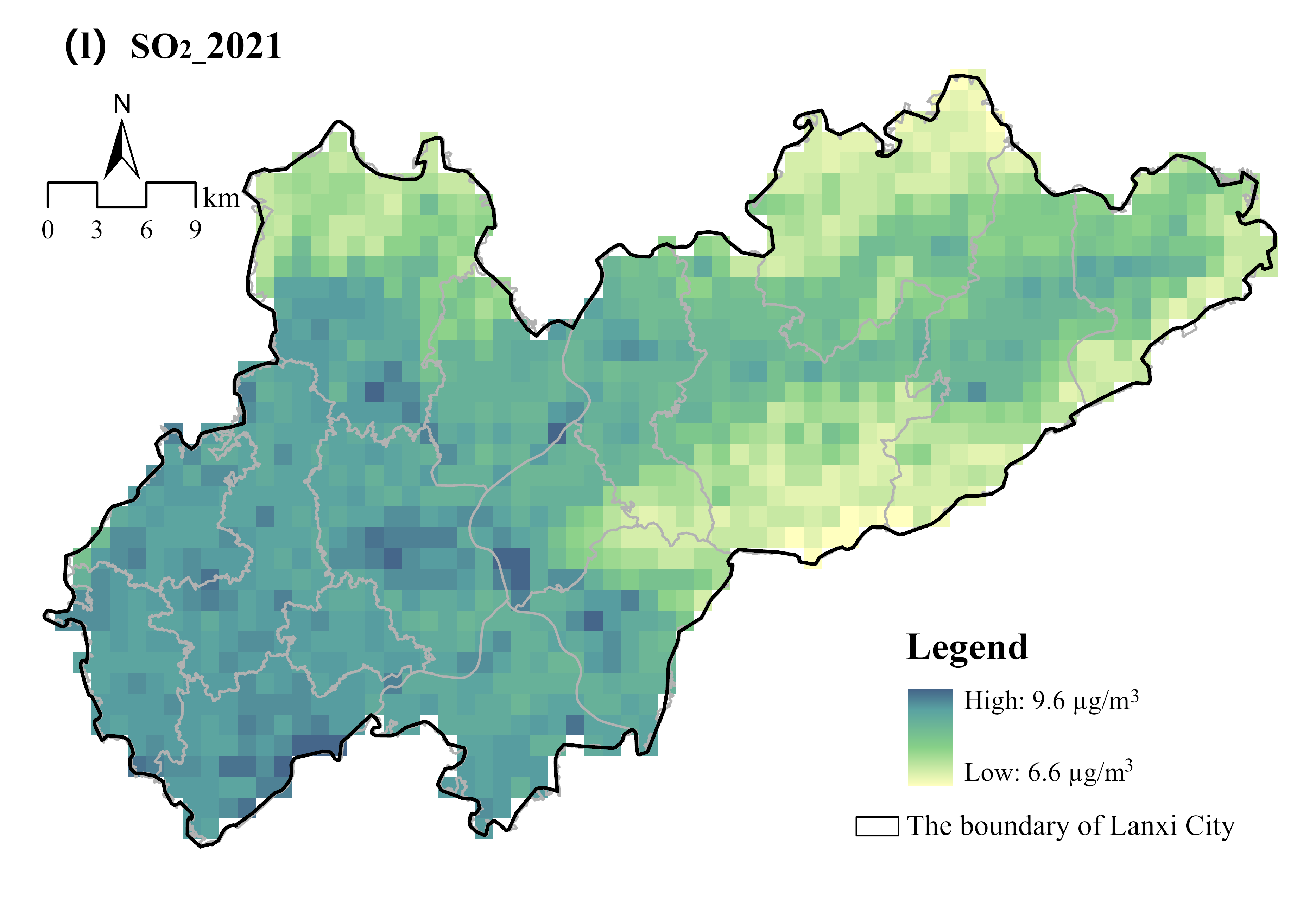


**Fig. S1.** Spatial distribution of annual average concentrations of PM₂.₅, NO_2_, O₃ and SO_2_ in Lanxi City during the study period.
